# Chloracidobacterium validum sp. nov., a thermophilic chlorophotoheterotrophic bacterium of the phylum Acidobacteriota from an alkaline hot spring microbial mat, represents Chloracidobacterium gen. nov., Chloracidobacteriaceae fam. nov. and Chloracidobacteriales ord. nov.

**DOI:** 10.1099/ijsem.0.007003

**Published:** 2026-01-19

**Authors:** Mohit Kumar Saini, Steven B. Kuzyk, Cristian Villena-Alemany, Sarah Kirstein, Jacqueline Wolf, Meina Neumann-Schaal, Shin Haruta, Satoshi Hanada, Michal Koblížek, Vera Thiel, Marcus Tank, Donald A. Bryant†

**Affiliations:** 1 Department of Biological Sciences, Tokyo Metropolitan University, 1-1 Minami-Osawa, Hachioji, Tokyo 192-0397, Japan; 2 Laboratory of Anoxygenic Phototrophs, Institute of Microbiology CAS, 37901 Třeboň, Czechia; 3 Leibniz Institute DSMZ – German Collection of Microorganisms and Cell Cultures GmbH, Inhoffenstraße 7B, 38124 Braunschweig, Germany; 4 Department of Biochemistry and Molecular Biology, The Pennsylvania State University, University Park, PA 16802, USA; 5 Department of Chemistry and Biochemistry, Montana State University, Bozeman, MT 59717, USA

**Keywords:** anoxygenic photosynthesis, *Chloracidobacteriaceae*, *Chloracidobacteriales*, chlorophotoheterotroph, microaerophile, thermophile

## Abstract

A novel chlorophototrophic bacterium, strain BV2-C^T^, was isolated from a microbial mat (~40 °C) at Rupite hot springs, Bulgaria. Cells were rod-shaped, Gram-negative, with numerous fimbriae/pili, but non-motile. Optimal growth occurred under micro-oxic conditions in the light (20–50 µmol photons m^−2^ s^−1^) at 45 °C (range 35–50 °C) and pH 7.2 (range pH 5.5–9.0). Bacteriochlorophyll *c*, bacteriochlorophyll *a*_P_, echinenone and *γ*-carotene were the major pigments in cells capable of producing chlorosomes and type-1 homodimeric reaction centres. Dense liquid culture appeared greenish-brown, with cells forming small clumps and aggregates. Strain BV2-C^T^ grows photoheterotrophically using organic compounds as carbon source (i.e. a mixture of 20 proteinogenic amino acids or peptone) and thioglycolate as reduced sulphur source. Strain BV2-C^T^ obligately required branched-chain amino acids (l-isoleucine, l-leucine and l-valine) and l-lysine for growth. It is closely related to *Chloracidobacterium thermophilum* and ‘*Candidatus Chloracidobacterium aggregatum*’ with 16S rRNA gene sequence identities of 96.97 and 97.78%, respectively. The genome of strain BV2-C^T^ comprises two circular chromosomes of 2,659,040 and 1,000,103 bp (total genome, 3.65 Mb) and G+C content of 59.9 mol%. Phenotypic, phylogenetic and genomic analyses demonstrated that *Chloracidobacterium validum* strain BV2-C^T^ (=DSM 113832^T^=JCM 39534^T^) represents a novel species of the genus ‘*Candidatus* Chloracidobacterium’, which we hereby propose as *Chloracidobacterium* gen. nov. with *Chloracidobacterium validum* sp. nov. as the type species. Based on phylogenomic analyses, we also propose a new family, *Chloroacidobacteriaceae* fam. nov., and a new order, *Chloracidobacteriales* ord. nov., within the class *Blastocatellia*. We further propose the name ‘*Ca. C. aggregatum*’ for pro-valid publication with the genome sequence of strain E as nomenclatural type.

## Introduction

Members of the phylum *Acidobacteriota* Thrash and Coates 2021, with its first isolate *Acidobacterium capsulatum* strain 161 obtained from acidic mineral environments [1], are ubiquitously distributed in diverse habitats, including non-acidic soils [2,4], the rhizosphere [5,6], sponges [7], deep-sea plankton or sediments [8,9] and caves [10]. They are also found in extreme habitats, including hot springs [11,13], acidic mining lakes [14] and uranium-contaminated soils [15]. Collectively, these bacteria have a phylogenetic diversity nearly as broad as the *Pseudomonadota* [16]. The phylum *Acidobacteriota* Thrash and Coates 2021 [17,19] encompasses 26 subdivisions clustered in 15 classes [20], of which only 5 are validly named under the International Code of Nomenclature of Prokaryotes (ICNP) and contain cultured representatives (*Blastocatellia* Pascual *et al*. 2016 [21,22], *Terriglobia* Thrash and Coates 2022 [23,24], *Vicinamibacteria* Dedysh and Yilmaz 2018 [20,25], *Thermoanaerobaculia* Dedysh and Yilmaz 2018 [20,25] and *Holophagae* Fukunaga *et al*. 2008 [26,27]).

Bioinformatic analyses of metagenomic sequences provided initial evidence for the occurrence of a novel chlorophototroph with homodimeric type-1 reaction centres and chlorosomes in microbial mats associated with the alkaline, siliceous hot springs, Octopus and Mushroom Spring, in Yellowstone National Park (YNP), WY, USA [28]. Initially established as an enrichment culture, *Chloracidobacterium thermophilum* strain B was shown to belong to the acidobacteria with the pro-validly published name ‘*Candidatus C. thermophilum*’ [28,29]. Leveraging information gleaned from a consensus (non-clonal) genome sequence [30] and information from diel metatranscriptomic analyses of the organism *in situ* [31], Tank and Bryant described strain B as an axenic isolate with defined growth medium in 2015 [32,33]. *C. thermophilum* strain B is an oligotroph with auxotrophies for branched-chain amino acids (l-leucine, l-isoleucine and l-valine), l-lysine and vitamin B_12_, further requiring reduced sulphur compounds as a sulphur source, with its growth stimulated by the addition of bicarbonate [32,34]. Although *C. thermophilum* is unable to synthesize branched-chain amino acids, it nonetheless can degrade them as well as other amino acids and use them as a carbon, nitrogen and sulphur source [30,32,34]. *C. thermophilum* is additionally a thermophilic microaerophile that obligately requires traces of oxygen to synthesize chlorophylls (Chls) and the amino acid tyrosine from phenylalanine, but is unable to tolerate atmospheric levels of oxygen [30,32,34].

Besides Octopus and Mushroom Springs in YNP, organisms similar to *Chloracidobacterium* have been found in microbial mats associated with other circumneutral and alkaline hot springs in YNP [35] and the Ojo Caliente hot springs in New Mexico, USA [36]. Related bacteria have further been detected during surveys of hot spring microbial mats around the world, for instance, in Tibet [37,38], Thailand [39], Japan [40] and Bulgaria [41]. Consequently, different species and/or ecotypes of *Chloracidobacterium* are suggested to be associated with the microbial mats of alkaline and circumneutral hot springs worldwide [34,42]. Based on the characterization of nine representing isolates, *Chloracidobacterium* is presently the sole genus of the phylum *Acidobacteriota* whose members perform Chl-dependent phototrophy (i.e. chlorophototrophy) [20,32, 34, 41]. While affiliated with the class *Blastocatellia* (formerly subdivision 4), this genus is only distantly related to the other genera within the class [20,32, 41]. Although it has been assumed that these bacteria potentially define a novel order and family [20], the previously limited number of effectively published names within this genus inhibited the description of a corresponding order and family.

The development of a defined growth medium for *C. thermophilum* [32,33] allowed for several additional strains of *Chloracidobacterium* species to be isolated from alkaline hot spring environments. Saini *et al.* [41] recently compared the genomes of nine axenic strains of *Chloracidobacterium*, of which eight strains were isolated from hot springs in YNP and one strain from Rupite hot spring in Bulgaria. Comparative bioinformatics suggested that these strains represent three species, *C. thermophilum*, *C. aggregatum* and *C. validum* [41]. Currently, the names ‘*Chloracidobacterium*’ and ‘*C. thermophilum*’ are only effectively and not validly published due to restrictions on the availability of the type strain B [32]. This prevented a valid publication of novel higher taxa (fam. nov. and ord. nov.). We here propose the names *Chloracidobacterium validum* sp. nov. (with strain BV2-C^T^ as type strain) as well as *Chloracidobacterium* gen. nov. (with *C. validum* as type species), *Chloracidobacteriaceae* fam. nov. and *Chloracidobacteriales* ord. nov., within the class *Blastocatellia* Pascual *et al*. 2016, phylum *Acidobacteriota* Thrash and Coates 2021 [25,43]. We further propose the name ‘*Candidatus Chloracidobacterium aggregatum*’ for pro-valid publication with the genome sequence of strain E as nomenclatural type and emend the description of the pro-validly published species name of ‘*Ca. C thermophilum*’ Bryant *et al.* 2007 [28,29].

## Sampling site, isolation and purification

Strain BV2-C^T^ was isolated from a Rupite hot spring microbial mat, located in the Sandanski-Petrich basin, Blagoevgrad Province, SW, Bulgaria (41° 21′ N 23° 14′ E). Situated in the Baba Vanga sanctuary, this spring feeds a cascade of six thermal water pools (BV1–BV6) connected by narrow channels (BV C1–5) (Fig. S1, available in the online Supplementary Material; also see [44]). A microbial mat sample was collected in April 2015 from pool BV2 at a site with a temperature of ~40 °C and pH 7.5–8.0 (Fig. S1). For isolation and purification of strain BV2-C^T^, mat sample material was incubated in agar plates containing *C. thermophilum* midnight (CTM) medium (Text S1) in the same way as previously described in detail [33,41]. The isolation and purification of strain BV2-C^T^ was performed under micro-oxic conditions (2–10% oxygen) with continuous illumination provided by incandescent lamps (60 W, 20–50 µmol photons m^−2^ s^−1^) at 45 °C. Axenicity was initially assessed by epifluorescence phase-contrast microscopy (Nikon Eclipse E600, Nikon, Japan) as described [33] and later verified by genome analysis (see below). Once axenic, colonies were transferred into freshly prepared liquid CTM medium to obtain larger amounts of bacterial biomass for further characterization. *C. thermophilum* strains B and D, as well as *C. aggregatum* strains E, N, S, A, 2 and MS40/45, were isolated from samples of hot spring associated microbial mats, recovered from Mushroom and Octopus Springs in YNP, WY, USA. Samples were taken under National Park Service (NPS Scientific Research and Collecting Permit number #YELL-SCI-0129 [33,41].

## Phenotypic and biochemical characterization

**Fig. 1.**
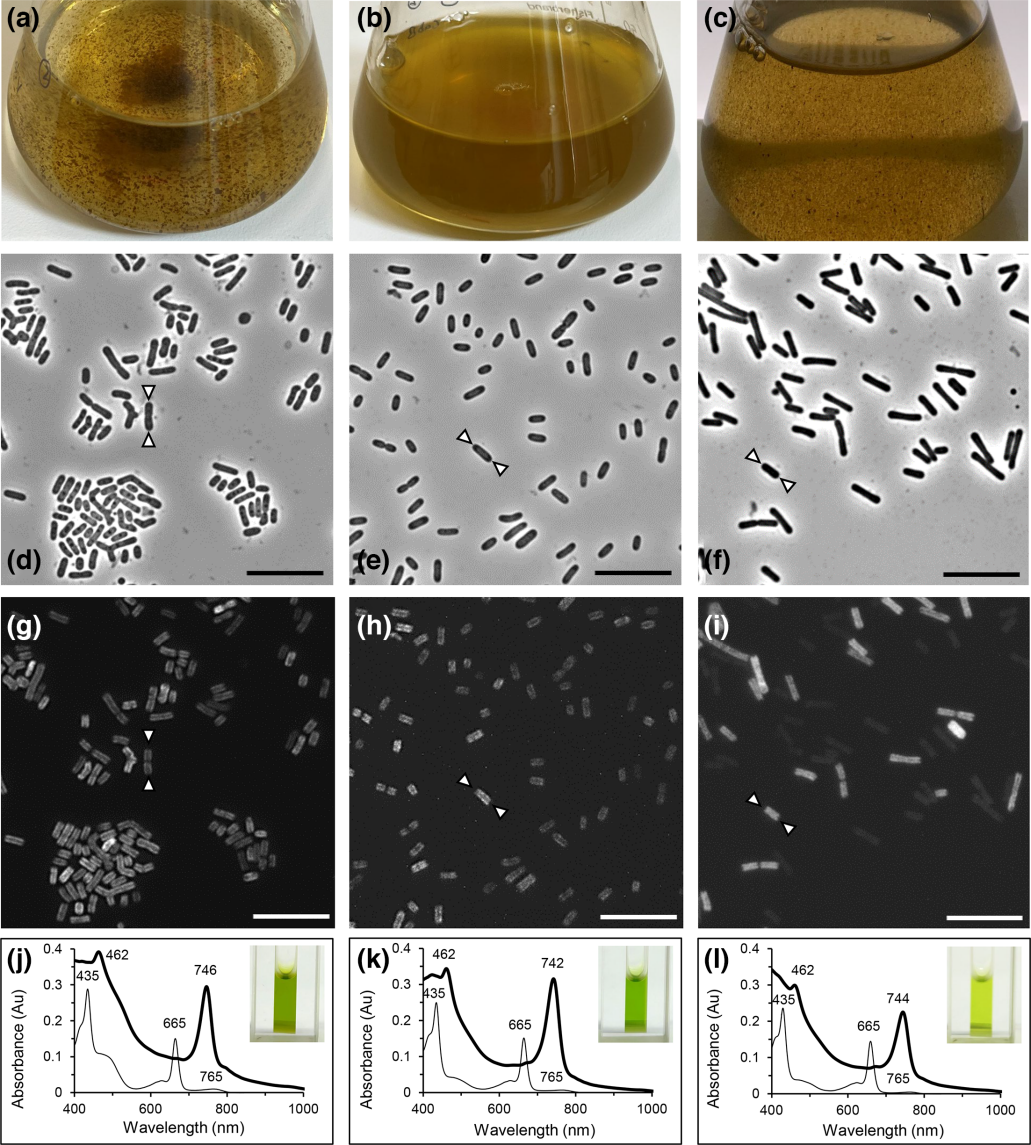
Dense greenish-brown liquid cultures of (a) aggregating *C. validum* strain BV2-C^T^, (b) homogenous *C. thermophilum* strain B and (c) aggregating ‘*Ca. C. aggregatum*’ strain E. Phase-contrast microscopic images, showing the rod-shaped cell morphology of (d) strain BV2-C^T^, (e) strain B and (f) strain E, are complemented by epifluorescence microscopy revealing BChl autofluorescence, but not at the end of the cells (indicated by white arrows) (g)–(i) of the three respective strains. Scale bars are 10 µm. Whole cell (bold line) and extracted pigment (thin line) absorbance spectra of (j) strain BV2-C^T^, (k) strain B and (l) strain E detailing maxima with green pigment extracts shown as insets.

Liquid cultures of strain BV2-C^T^ were similar in colour to strain B, i.e. greenish-brown (Fig. 1a, b). Strain BV2-C^T^ formed visible cellular aggregates in liquid culture (Fig. 1a), similar to cultures of *C. aggregatum* strain E (Fig. 1c), and was in contrast to the homogenous cell suspensions of *C. thermophilum* strain B (Fig. 1b). Gram-negative cells of strain BV2-C^T^ were rod-shaped, ~2–4 µm long and 0.8–1.0 µm wide (Fig. 1d), similar to both strains B and E (Fig. 1e, f). Gram-negative cellular membranes with inner peptidoglycan wall were confirmed via transmission electron microscopy (TEM) of thin sections (Fig. S3), prepared as described [45]. TEM of negatively stained cells was performed as described previously [46] and revealed numerous pili or fimbriae, which were larger in diameter and possibly more rigid in strain BV2-C^T^ than those observed on *C. thermophilum* strain B (Fig. S2). Since flagella were not detected and twitching motility was not observed, these fimbriae are suggested to be involved in attachment, as shown by the clumping of some cultures. Epifluorescence microscopy using a Nikon Eclipse E600 microscope equipped with monochromatic CMOS camera (Orcaflash 4.0, Hamamatsu) and XPS-100 xenon lamp further revealed bacteriochlorophyll (BChl) autofluorescence of all three *Chloracidobacterium* spp. using 445 nm (+/− 30 nm) excitation filter and 700 nm long pass emission filter (Semrock, Rochester, NY, USA) (Fig. 1g–i). Cells of strain BV2-C^T^ exhibited autofluorescence from BChl *c*, which was not distributed evenly throughout the cell but was more intense near the lateral cell walls but not at the poles of the cells (Fig. 1g). This distribution has previously been described for *C. thermophilum* strain B, attributed to chlorosomes placement only along the lateral cell walls [32,33, 47]. Taken together, the pattern of autofluorescence and the presence of genes encoding both BChls *a* and *c* biosynthesis as well as chlorosome envelope proteins [41,47], this provides strong presumptive evidence for the existence of chlorosomes in strain BV2-C^T^.

### Pigment analyses

The *in vivo* (photosynthetic whole cells) and *in vitro* (organic solvent-extracted pigments) spectra of *Chloracidobacterium* spp. strains were measured via a PerkinElmer Lambda 365+ (Rodgau, Germany) within the range of 400–1,000 nm. Sample preparation for measurement of the *in vivo* and *in vitro* spectra was performed as previously described in 70% glycerol solution and acetone:methanol (7:2), respectively [48]. The *in vivo* absorption spectrum of strain BV2-C^T^ showed peaks at 746 and 462 nm, suggesting the predominance of BChl *c* bound in chlorosomes, which was similar to the absorption spectra of strain B and strain E (Fig. 1j–l). Moreover, all *Chloracidobacterium* strains contain highly similar *in vivo* spectra with peaks between 742 and 746 nm (Fig. S4). These small deviations in the maxima may suggest minor conformational changes for each photosynthetic complex. In comparison, the *in vitro* spectrum of strain BV2-C^T^ was identical to the other strains and revealed predominant maxima at 665 and 435 nm (Fig. 1j–l), which represent the Q_y_ band and Soret band of BChl *c*, respectively. A minor peak at 765 nm further matches trace levels of BChl *a* in all strains. Complemented by previous work [41], strain BV2-C^T^ and other *Chloracidobacterium* spp. contain echinenone, canthaxanthin and *γ*-carotene as predominant carotenoids, and the major BChls are BChls *c* and *a*_P_ (Table 1). Further, minor (B)Chls, Chl *a*_PD_ and Zn-BChl *a*_P_′, are also present in functional type-1 reaction centres as in *C. thermophilum* strain B [41,49].

**Table 1.** Characteristics of *Chloracidobacterium* spp. compared to other members of the class *Blastocatellia.* All strains are Gram-negative and predominantly rod-shaped. NP, not present; ND, not determined; AA, Amino acids; req., requirement; –, no genome sequences were currently available; strain BV2-C^T^, *C. validum*; strain B, *C. thermophilum*; strain E, *C. aggregatum*.

Characteristic	Order	*Chloracidobacteriales*			*Blastocatellales*‡						
	Family	*Chloracidobacteriaceae*			*Pyrinomon* *-adaceae*	*Arenimicrobiaceae*		*Blastocatellaceae*			
	Genus	Chloracidobacterium*			*Pyrinomonas*	*Arenimicrobium*	*Brevitalea*	*Blastocatella*	*Aridibacter*	*Tellurimicrobium*	*Stenotropho* *-bacter*
	Strain	BV2-C^T^	B*	E†							
**Cell shape**		Rod	Rod	Rod	Rod	Ovoid to rod	Rod	Sphere to rod	Rod	Rod	Rod
**Cell** **size** **(length×** **width)** **[µm]**		2–4×0.8–1.0	~2.5×0.8–1.0	2–4×~0.8	1.0–4.0×0.3–0.6	1.0–2.3×0.7–1.0	0.9–2.2×0.6–0.9	0.8–12.0×0.8–0.9	1.5–3.0×0.6–0.9	0.8–2.3×0.3–0.7	0.8–2.5×0.3–0.7
**Cell division**		Binary fission	Binary fission	Binary fission	Binary fission	Binary fission	Binary fission	Binary fission/budding	Binary fission	Binary fission	Binary fission
**Motility**		No	No	No	No	No	No	Yes	No	No	No
**Temp. (°C) optimum (range)**		45 (35–50)	~52 (44–61)	~50 (39–58)	65 (50–69)	28–44.6 (8–44.6)	35–45 (11–52.5)	30–35 (14–40)	24–44 (8–47)	34–37 (14–43)	29–41 (16–41)
**pH optimum (range)**		7.2 (5.5–9.0)	7 (5.5–9.5)	~7.0 (5.5–9)	6.5 (4.1–7.8)	5.5–7.0 (3.5–9.5)	5.4–7.0 (4.7–9.0)	5.0–7.5 (4.0–10.0)	5.5–9.0 (3.5–10.0)	5.6–6.5 (4.7–8.4)	5.5–8.0 (4.5–10.0)
**Oxygen requirement**		Micro -aerophilic	Microaerophilic	Microaerophilic	Aerobic	Aerobic	Aerobic	Aerobic	Facultatively anaerobic to aerobic	Aerobic	Aerobic
**Colour of cell suspension**		Greenish-brown	Greenish-brown	Greenish-brown	White-semitransparent	Orange-yellow	White	Orange-pink	White/yellow/pink	White (bright pinkish hue)	Pink
**Cell suspension in liquid**		Aggregates	Homogenous	Aggregates	Aggregates	Aggregates	Aggregates	No aggregates	Aggregates/no aggregates	Small aggregates	No aggregates
**Metabolism**		Anoxygenic chlorophoto -heterotroph	Anoxygenic chlorophoto -heterotroph	Anoxygenic chlorophoto -heterotroph	Chemoorgano -troph	Chemoorgano -troph	Chemoorgano -troph	Chemoorgano -troph	Chemoorgano -troph	Chemoorgano -troph	Chemoorgano -troph
**(Bacterio)-Chlorophylls major (minor)**		BChl *c*, BChl *a*_P_ (Chl *a*_PD_, Zn-BChl *a*_P_′)	BChl *c*, BChl *a*_P_ (Chl *a*_PD_, Zn-BChl *a*_P_′)	BChl *c*, BChl *a*_P_ (Chl *a*_PD_, Zn-BChl *a*_P_′)	NP	NP	NP	NP	NP	NP	NP
**Major Carotenoids**		Echinenone, canthaxanthin, *γ*-carotene	Echinenone, canthaxanthin, *γ*-carotene	Echinenone, canthaxanthin, *γ*-carotene	nd	nd	nd	nd	nd	nd	nd
**Vitamin B**_**12**_ **req.**		No	Yes	(Yes)§	nd	nd	nd	nd	nd	nd	nd
**Branched-chain AA req.**		Yes	Yes	Yes	nd	nd	nd	nd	nd	nd	nd
**l** **-Lysine req.**		Yes	Yes	Yes	nd	nd	nd	nd	nd	nd	nd
**Methionine synthase**		MetH; no MetE	MetH; no MetE	MetH; no MetE	nd	–	–	–	–	–	–
**Ribonu** **-cleotide** **reductase**						–	–	–	–	–	–
NrdAB (Class Ia)		Present	Present	Present	nd	–	–	–	–	–	–
NrdJ (Class II)		Absent	Present	Absent	nd	–	–	–	–	–	–
NrdD-like paralog (Class III)		Present (no NrdG)	Present (no NrdG)	Present (no NrdG)	nd	–	–	–	–	–	–
**Mg-** **protopor** **-phyrin** **IX** **mono** **-methyl** **ester oxidative** **cyclase**		BchE (O_2_-independent) AcsF (O_2_-dependent)	BchE (O_2_-independent) AcsF (O_2_-dependent)	BchE (O_2_-independent) (AcsF: absent)	nd	–	–	–	–	–	–
**Genome size (Mb)**‖		3.66	3.75	3.77	~3.80	–	–	–	~3.80–4.40¶	–	–
Number of chromosomes		2	2	2	nd	–	–	–	–	–	–
Chromosome sizes (Mb)		2.66+1.0	2.70+1.05	2.67+1.09	nd	–	–	–	–	–	–
**Total G+C (mol%)**		59.5	61.31	62.18	59.36	66.9	54.7–55.9	46.5	46.5–53.2	59.4	56.8–58.5

*Properties of *C. thermophilum* strain B have been described previously [32,34].

†Properties of *C. aggregatum* strains have been described [33,41].

‡Properties of *Blastocatellales *genera *Pyrinomonas*, *Arenimicrobium* and *Brevitalea* [69], *Blastocatella* [70], *Aridibacter* [71], *Tellurimicrobium* and *Stenotrophobacter* [21,58], as described.

§Vitamin B_12_ stimulates growth but essentiality not tested.

‖Sizes, accession numbers, synteny and other information are available in Saini *et al.* [41].

¶Value is taken from the MAG identified as *A. famidurans*; whole genome of the type strain is not sequenced yet.

### Physiological and biochemical analyses

Unless otherwise specifically stated, all physiological and biochemical experiments involved strain BV2-C^T^ grown using CTM medium in 100 ml Erlenmeyer flasks filled with 80 ml of medium at 45 °C and pH 7.2 under continuous illumination from a 60 W tungsten bulb (∼20–50 µmol photons m^−2^ s^−1^). Due to its oligotrophy, cultures were repetitively fed with aliquots of a 20 proteinogenic amino acid mixture (300–500 mg l^−1^ combined final concentration) at intervals of 3–4 days to obtain higher bacterial biomass yields per culture. The detailed description of CTM medium composition [33] and its preparation is described in Text S1. Strain BV2-C^T^ was able to grow photoheterotrophically in CTM medium under micro-oxic conditions utilizing organic compounds (i.e. a mixture of the 20 proteinogenic amino acids and/or peptone) as carbon source and thioglycolate as reduced sulphur source, similar to all known *Chloracidobacterium* isolates but unlike any member of *Blastocatellales* (Table 1). Amino acids could be utilized not only as a carbon source but also served as a supply of nitrogen and reduced sulphur (l-methionine and l-cysteine). Photoautotrophic growth was not observed when incubated with bicarbonate as the only carbon source, which was supported by an absence of carbon fixation genes in all nine sequenced strains [41]. Further, all of the analysed genomes lacked nitrogen fixation-related genes, indicating the strains depend on other nitrogen sources than dinitrogen as mentioned above. Strain BV2-C^T^ did not replicate under dark conditions and thus is an obligate chlorophototroph. It neither grew under a fully anoxic environment nor under atmospheric oxygen levels on solid or in liquid medium. When inoculated into agar deeps, strain BV2-C^T^ grew in the micro-oxic zone (2–10% oxygen) similar to *C. thermophilum* strain B (Fig. S5). Thus, strain BV2-C^T^ is a microaerophile with a similar oxygen tolerance to *C. thermophilum* strain B. Growth at different pH values was tested in CTM medium buffered with 20 mM MES (pH 5–6.5), HEPES (pH 7–7.5), Tricine (pH 8–8.5) or CHES (pH 9–9.5). The optimum pH for growth was pH 7.2–7.5 (range pH 5.5–9.0), similar to the optimum value observed for *C. thermophilum* strain B [32,33] or *C. aggregatum* strain E (Table 1). Strain BV2-C^T^ grew optimally at 45 °C (range 35–50 °C), characterizing it as a moderately thermophilic bacterium. This optimum was lower than that of *C. thermophilum* strain B (Table 1), yet both are in accordance with the hot spring temperatures from which each was isolated.

Vitamin B_12_ auxotrophy was shown for *C. thermophilum* strain B in previous studies [32,33]. The vitamin requirements for strain BV2-C^T^ (with *C. thermophilum* strain B as control) were tested by growing both strains in CTM medium (no peptone version) amended either (1) with vitamin solution A (a mixture of 13 vitamins; see Text S1), (2) vitamin solution B (vitamin B_12_), (3) with both vitamin solutions A and B or (4) without any vitamin source. In contrast to *C. thermophilum* strain B, growth of strain BV2-C^T^ occurred independently of added vitamin B_12_. Growth occurred when cells were provided either with vitamin solution A (mixture of 13 vitamins) or vitamin solution B (vitamin B_12_), whereas strain BV2-C^T^ was inhibited without a vitamin source (Fig. S6). Genomic sequence analysis indicates that strain BVC-2^T^ lacks a complete set of genes for adenosylcobalamin biosynthesis, but a salvage pathway is present that should allow cells to utilize vitamin B_12_ derivatives obtained from the environment [30,41]. Vitamin B_12_ is usually used by cells as a cofactor for several important enzymes: cobalamin-dependent methionine synthase (MetH) [50], vitamin B_12_-dependent ribonucleotide reductase (NrdJ) [51,52], vitamin B_12_-dependent methyl-malonyl CoA mutase [53] and oxygen-independent magnesium-protoporphyrin IX monomethyl ester oxidative cyclase (BchE) [54]. Differences in the distribution of the genes encoding these enzymes among the *Chloracidobacterium* spp. could explain the variation in phenotypes (Table 1). For example, genomes of all three species contain Class-Ia ribonucleotide reductase genes (*nrdAB*), while only *C. thermophilum* strain B and one *C. aggregatum* strain (MS 40/45) have Class-II cobalamin-dependent ribonucleotide reductase genes (*nrdJ*), which could function under anoxic conditions when vitamin B_12_ is present in the environment. Concerning magnesium-protoporphyrin IX monomethyl ester oxidative cyclase: all strains have cobalamin-dependent *bchE* [54], yet most *C. aggregatum* strains lack *acsF* (strain MS40/45 is an exception) (Table 1).

Like *C. thermophilum* strain B [30], the genome of strain BV2-C^T^ lacks the genes required to synthesize branched-chain amino acids (l-isoleucine, l-leucine and l-valine) and l-lysine but contains genes needed to produce the enzymes capable of degrading such amino acids [41]. To confirm this metabolic inference, strain BV2-C^T^ and *C. thermophilum* strain B were grown in CTM medium with specific mixtures of amino acids (Fig. S7). Strain BV2-C^T^ and strain B neither grew when all 20 amino acids and/or peptone were omitted from the growth medium nor when supplied a mixture of 16 amino acids (all except branched-chain amino acids and l-lysine). However, both strains could grow when cultures were provided with all proteinogenic branched-chain amino acids and l-lysine; l-lysine alone did not support growth of either strain (Fig. S7). These results indicate that, like *C. thermophilum* strain B, strain BV2-C^T^ obligatorily requires branched-chain amino acids as well as l-lysine (Table 1). Peptone can serve as a source of these necessary (and other) amino acids, but the biomass yield was lower when only peptone was provided (Fig. S7).

### Chemotaxonomy analyses

Cellular respiratory quinones, fatty acids and polar lipids of all strains of *Chloracidobacterium* were quantified as described previously [55]. MK-8(H_2_) was their major menaquinone, whereas MK-8(H_4_) made up a small fraction in each (Table S1). While minor strain-to-strain deviations in relative abundances in fatty acids were found between every representative (Table S1), clear patterns emerged (Table 2). The predominant fatty acid of *Chloracidobacterium* is iso-C_15:0_, similar to all other members of the class *Blastocatellia*, followed by C_16:0_, iso-C_16:0_, C_18:0_ and iso-diabolic acid, the last of which is also common among *Acidobacteriota* phyla members [56]. The polar lipids diacylglyceryl-hydroxymethyl-*N*,*N*,*N*-trimethyl-beta-alanine (DGTA) and lyso-DGTA were unique to *Chloracidobacterium*, while phosphatidylethanolamine (PE), phosphatidyl-*N*-monomethylethanolamine (PME) and phosphatidylcholine (PC) can be found in many other taxa of the class *Blastocatellia* (Tables 2 and S2). Although phosphatidylglycerol (PG) was previously reported in *C. thermophilum* strain B [32,34], it was not detected in our recent experiments (Table S2). However, this polar lipid was found at trace levels in a few *Chloracidobacterium* strains.

**Table 2.** Cellular fatty acid, polar lipids and menaquinone analysis of *Chloracidobacterium* spp. compared to other members of the class *Blastocatellia* ND, Not determined.

Order	Family	Genus (strains)		Polar lipids	Major fatty acids	Major quinone
*Chloracidobacteriales*	*Chloracidobacteriaceae*	*Chloracidobacterium*	(BV2-C^T^)	DGTA, lyso DGTA, PE, PME	iso-C_15:0_, C_16:0_, iso-C_16:0_, C_16:0_, iso-dibolic acid	MK-8(H_2_)
			(B)	DGTA, lyso DGTA, PE, PME	iso-C_15:0_, C_16:0_, iso-C_16:0_, C_16:0_, iso-dibolic acid	MK-8(H_2_)
			(E)	DGTA, lyso DGTA, PE, PME	iso-C_15:0_, C_16:0_, iso-C_16:0_, C_16:0_, iso-dibolic acid	MK-8(H_2_)
*Blastocatellales*	*Pyrinomonadaceae*	*Pyrinomonas*		PE, PC	iso-C_15:0_, iso-C_17:0_, iso-C_19:0_, iso-C_21:0_	MK-8
	*Arenimicrobiaceae*	*Arenimicrobium*		DPG, PE, PC, PI	iso-C_15:0_, iso-C_17:0_, anteiso-C_17:0_, iso-C_15:1_ H/C_13:0_ 3-OH	MK-8
		*Brevitalea*		DPG, PE, PC, PI, Unid GPL	iso-C_15:0_, iso-C_17:0_, anteiso-C_17:0_, iso-C_15:1_ H/C_13:0_ 3-OH	MK-8, MK-7
	*Blastocatellaceae*	*Blastocatella*		nd	iso-C_16:1_ *ω7c*/C_15:0_ 2-OH, iso-C_15:1_ H/C_13:0_ 3-OH, iso-C_16:0_, iso-C_17:1_ *ω9c*	MK-8
		*Aridibacter*		DPC, PC, PE, PG	iso-C_15:0_, C_13:0_ 3-OH/iso-C_15:1_ H, C_16:1_ *ω7c*/C_16:1_ *ω6c*, anteiso-C_17:0_, anteiso-C_15:0_	MK-8
		*Tellurimicrobium*		DPG, PE, PC, PG, Unid GPL	C_18:1_ *ω7c*/C_18:1_ *ω6c*, iso-C_15:0_, C_16:1_ *ω7c*/C_16:1_ *ω6c*, iso-C_15:1_ H/C_13:0_ 3-OH	MK-8
		*Stenotrophobacter*		DPG, PE, PC, PG	iso-C_15:0_, C_16:1_ *ω7c*/C_16:1_ *ω6c*, iso-C_15:1_ H/C_13:0_ 3-OH	MK-8

DPG, Diphosphatidylglycerol; PC, phosphatidylcholine; PE, phosphatidylethanolamine; PG, phosphatidylglycerol; PI, phosphatidylinositol; PME, phosphatidyl-N-monomethylethanolamine; Unid GPL, unidentified glycophospholipid.

## Phylogenetic and phylogenomic analyses

**Fig. 2.**
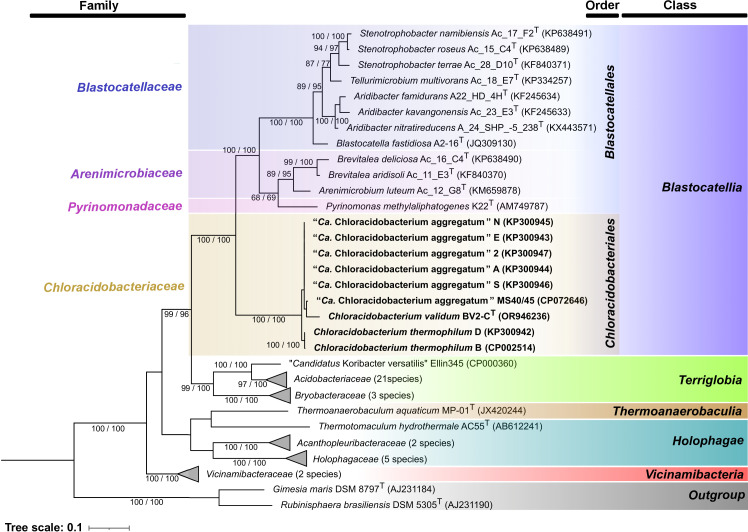
Inferred maximum-likelihood phylogenetic tree based upon 16S rRNA gene sequences of selected members of the *Acidobacteriota*. The branches are scaled in terms of the expected number of substitutions per site. The numbers above the branches indicate support values when larger than 60% from maximum-likelihood (left) and maximum parsimony (right) analysis based upon 1,000 bootstrapping replicates. This tree provides strong support for the creation of a new family, *Chloracidobacteriaceae* (orange-yellow shading), and new order, *Chloracidobacteriales*, within the class *Blastocatellia* (blue shading) and within the phylum *Acidobacteriota*. The five presently recognized classes, *Blastocatellia* (blue shading), *Terriglobia* (green shading), *Thermoanaerobaculia* (brown shading), *Holophagae* (cyan shading) and *Vicinamibacteria* (red shading), are shown at the right. The three currently recognized families of the order *Blastocatellales* are shown by different shading: *Blatocatelliaceae* (blue), *Arenimicrobiaceae* (orchid) and *Pyrinomonadaceae* (magenta). Two strains from the phylum *Planctomycetota* were used as outgroup. Accession numbers and taxonomic assignments for the organisms included in this tree are provided in Table S3.

The methods for DNA isolation, genome sequencing, assembly of circularized chromosomes, annotation, single-value whole-genome or 16S rRNA phylogenetic sequence comparisons were performed as previously described [41]. Fig. 2 shows an inferred maximum-likelihood phylogenetic tree based upon 16S rRNA gene sequences of selected members of *Acidobacteriota*, including representative strains from all five currently recognized classes of the phylum: *Blastocatellia*, *Terriglobia*, *Vicinamibacteria*, *Thermanaerobaculia* and *Holophagae* (Table S3). This phylogenetic tree revealed all *Chloracidobacterium* spp. form a clade that is most closely related to, but clearly separate and distinct from, other members of the order *Blastocatellales* within the class *Blastocatellia*. Although *Blastocatellia* presently includes two orders, *Blastocatellales* and ‘*Ca.* Frugalibacterales’ [20,57], where the latter is defined by uncultured organisms ‘GAL08’ found in neutral hot springs [57], *Chloracidobacterium* spp. clearly separate from both orders [57]. Moreover, *Chloracidobacterium* spp. strains are not closely related to any member of the three families *Pyrimonadaceae*, *Blastocatellaceae* and *Arenimicrobiaceae* of *Blastocatellales* (Fig. 2, Tables 3 and S3) [20,21, 58]. The most similar members of the class *Blastocatellia* are merely ~85% identical to the 16S rRNA gene sequence of strain BV2-C^T^ (Fig. 2), which is above the current threshold (78.5%) for class separation as well as for order separation (82%), but below the threshold for family delineation (86.5%) [59]. However, as the threshold for order-level separation (82%) is on the margin, a combined consideration of 16S rRNA, whole-genome phylogenomics (discussed below), as well as single-value whole-genome comparisons suggests *Chloracidobacterium* should be placed in a separate order. Thus, we propose that the collective *Chloracidobacterium* spp. represent a new order *Chloracidobacteriales*, which comprises a single family, *Chloracidobacteriaceae* (Fig. 2, Table 1).

**Table 3.** 16S rRNA identity, dDDH, ANI and AAI values for *Chloracidobacterium* spp. compared to other members of the class *Blastocatellia*

Organisms	*C. validum* BV2-C^T^				*C. thermophilum* B				*C. aggregatum* E			
	16S (%)*	dDDH (%)†	ANI (%)‡,§	AAI (%)‖	16S (%)	dDDH (%)	ANI (%)	AAI (%)	16S (%)	dDDH (%)	ANI (%)	AAI (%)
*C. validum* BV2-C^T^	100	100	100	100	96.83	19.10	75.22	80.20	97.41	19.20	75.38	80.30
*C. thermophilum* B	96.83	19.10	75.22	80.20	100	100	100	100	98.95	53.50	93.64	95.10
*C. aggregatum* E	97.41	19.20	75.38	80.30	98.95	53.50	93.64	95.10	100	100	100	100
*P. methylaliphatogenes* K22^T^	85.75	23.10	66.40	52.50	86.29	18.90	66.40	52.90	85.81	21.60	66.81	52.60
*A. famidurans* A22_HD_4H^T^ (isolate MAG.1 k127_1028782)¶	83.63	(21.8)	(65.6)	(49.9)	83.02	(19.7)	(65.98)	(50.1)	83.17	(22.0)	(65.5)	(50.1)
*Arenimicrobium luteum* Ac_12_G8^T^	84.91	nd#	nd	nd	85.13	nd	nd	nd	85.22	nd	nd	nd
*Brevitalea aridisoli* Ac_11_E3^T^	85.58	nd	nd	nd	85.13	nd	nd	nd	84.68	nd	nd	nd
*Blastocatella fastidiosa* A2-16^T^	83.35	nd	nd	nd	83.50	nd	nd	nd	83.37	nd	nd	nd
*Stenotrophobacter terrae* Ac_28_D10^T^	84.13	nd	nd	nd	84.01	nd	nd	nd	83.88	nd	nd	nd
*Tellurimicrobium multivorans* Ac_18_E7^T^	84.49	nd	nd	nd	84.83	nd	nd	nd	84.78	nd	nd	nd

*Five other *C. aggregatum* strains have 16S rRNA sequence identities that are 97.78–98.01% similar to *C*.* validum* strain BV2-CT and 98.39–98.99% similarity to *C*. *thermophilum* strain B. The 16S rRNA sequence of *C*.* thermophilum* strain D is 97.21% identical to *C*.* validum* strain BV2-CT and that is 99.93% identical to *C*. *thermophilum* strain B.

†Five other *C. aggregatum* strains had dDDH values that were 19.10–19.30% compared to *C. validum* strain BV2-CT and that were 44.60–53.60% compared to *C*. *thermophilum* strain B. *C*.* thermophilum* strain D has a dDDH value of 19.2% compared to *C*.* validum* strain BV2-CT and 83.7% compared to *C*. *thermophilum* strain B.

‡Five other *C. aggregatum* strains had ANI values that were 75.37–75.53% compared to *C. validum* strain BV2-CT and 91.55–93.73% compared to *C. thermophilum* strain B. The ANI value for *C*.* thermophilum* strain D is 75.2% compared to *C*.* validum* strain BV2-CT and 98.2% compared to *C*. *thermophilum* strain B.

§ANI was also calculated as ANIb [61] (data not shown), which produced very similar results to those shown above.

‖Five other *C. aggregatum* strains had AAI values that were 80.3–80.5% similar to *C*.* validum* strain BV2-CT and 93.4–95.1% similar to *C*. *thermophilum* strain B. *C*.* thermophilum* strain D has an AAI value of 80.2% compared to *C*.* validum* strain BV2-CT and 98.4% compared to *C*. *thermophilum* strain B.

¶Numbers in parentheses are for an MAG identified as *A. famidurans* as the type strain is not sequenced.

#Not determined, nd, because no genome sequences were currently available for these strains.

The assembled genome of strain BV2-C^T^ was previously deposited in NCBI (GCF_018304825.1) and annotated using the National Center for Biotechnology Information (NCBI) Prokaryotic Genome Annotation Pipeline (https://www.ncbi.nlm.nih.gov/refseq/annotation_prok/). The genome encodes 3,018 proteins and 53 RNAs, including 47 tRNAs, 3 identical rRNA operons (5S, 16S, 23S) and 3 ncRNAs. Due to differences in the annotation software used, this NCBI annotation differs slightly from the previously published annotation done in rapid annotation using subsystem technology [41]. The complete genome of strain BV2-C^T^ consists of two circular chromosomes with lengths of 2,659,040 and 1,000,103 bp, respectively, and shows a total length of 3,659,143 bp with a G+C content of 59.9 mol% (calculated from the sequence), similar to all other strains of the genus (Table 1). These values are comparable to the most similar members of *Blastocatellaceae*, *Pyrimonas methylaliphatogenes* K22^T^ and *Aridibacter famidurans* A22_HD_4H^T^ (genomic information taken from the MAG.1 k127_1028782), which have ~4.0 Mbp genomes with G+C content of 59–66 mol% (Table 1). The calculated average nucleotide identity (ANI) value between strain BV2-C^T^ and *C. thermophilum* strain B was 75.22%, while digital DNA–DNA hybridization (dDDH) relatedness was 19.10% (Table 3). In addition, ANI values between strain BV2-C^T^ and *C. aggregatum* strains were 75.3–75.5%, while the dDDH relatedness was 19.1–19.3%. Combined, these ANI and dDDH values fall far below the threshold values for species-level relatedness (95–96% ANI, 70% dDDH) [60,61] and together strongly support the proposal of strain BV2-C^T^ as a new species.

**Fig. 3.**
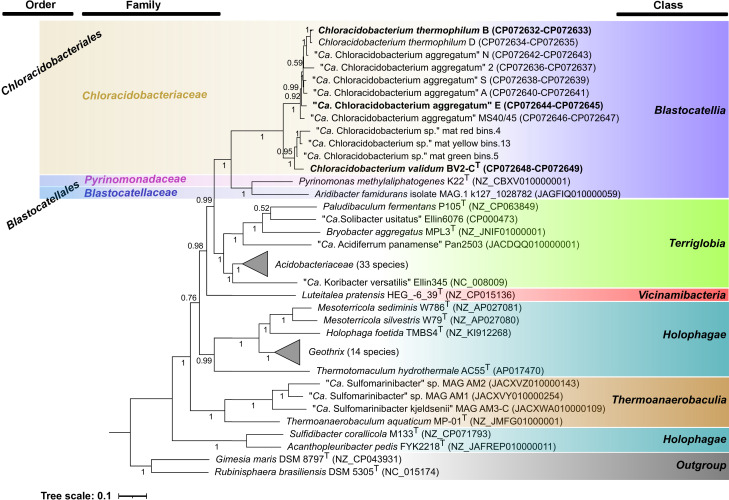
Inferred rooted maximum-likelihood phylogenomic tree of selected members of the phylum *Acidobacteriota*. The orange-yellow shading indicates the strains for the newly proposed order *Chloracidobacteriales* and newly proposed family *Chloracidobacteriaceae*. The *Chloracidobacterium* spp. strains are assigned to three species, *C. validum*, ‘*Ca. C. aggregatum*’ and *C. thermophilum*, as indicated by the strain names (also see Fig. 2 and text; also see [41]). The blue shading indicates two strains from the order *Blastocatellales* in the class *Blastocatellia* (blue shading at right). The other coloured bars at the right indicate strains belonging to the five presently characterized classes within the phylum *Acidobacteriota*. Two strains from the phylum *Planctomycetota* were used as outgroup. Accession numbers and taxonomic assignments for the organisms included in this tree are provided in Table S4, and a heat map depiction of the data is shown in Fig. S8

Phylogenomic analysis of *Acidobacteriota* genomes was performed with PhyloPhlAN 3.0 [62,63]. Two genomes from the bacterial phylum *Planctomycetota* were used as an outgroup (Table S4). PhyloPhlAN uses USEARCH [64] to detect 400 universally conserved and ubiquitous proteins, and it aligns them using muscle [65], concatenates the sequences and generates a maximum-likelihood tree with RAxML [66]. Taxonomic assignments and accession numbers for the organisms included in the analysis in Fig. 3 are provided in Table S4. Three metagenome assembled genomes (MAGs) were included in the phylogenomic analysis, which improved the resolution concerning the *Chloracidobacterium* species. Mat_green_bins.5 (JBDNCY000000000), mat_red_bins.4 (JBDNDF000000000) and mat_yellow_bins.13 (JBDNCT000000000) were reconstructed from samples taken from Rupite hot spring (41° 27′ 29″ N 23° 15′ 43″ E) metagenomes (red SAMN38396676, yellow SAMN38396664 and green SAMN38396669), which have previously been deposited under Bioproject PRJNA1041075. The phylum *Acidobacteriota* presently comprises five classes and a probable sixth class, ‘*Ca.* Polarisedimenticolia’ [20,67]. A seventh potential class, ‘*Ca.* Aminicenantia’, might possibly belong to the phylum *Acidobacteriota*, but these uncultured organisms could also form a candidate phylum; thus, both candidate taxa were not considered in this analysis [20,67, 68].

Based upon whole-genome analyses (Fig. 3), the nine isolates of the genus *Chloracidobacterium* belong to the class *Blastocatellia* (formerly subdivision 4 [20]). *Blastocatellia* presently includes the orders *Blastocatellales* and ‘*Ca.* Frugalibacterales’ [20,58], but like the 16S rRNA analyses described above (Fig. 2), the data in Fig. 3 support *Chloracidobacterium* spp. to represent a separate monophyletic order and family. The phylogenetic analysis further strongly supports the conclusions based on ANI, dDDH, tetranucleotide frequency and genome synteny [41] that the nine characterized strains define at least three species: *C. validum* strain BV2-C^T^, *C. thermophilum* strains D and B and *C. aggregatum* strains E, N, S, A, 2 and MS40/45. In addition, the phylogenomic tree (Fig. 3) and heat map analyses of the average amino acid identity (AAI) comparisons (Fig. S8) show three MAGs from Rupite hot spring forming a group with *C. validum* BV2-C^T^. Such additional data support the speciation of *C. validum* and attribute it as a steady member of the hot spring microbial mats at Rupite, Bulgaria [41]. Although strain MS40/45 was tentatively assigned to *C. aggregatum* [41], its species assignment remains ambiguous. This strain has a physiology similar to that of *C. thermophilum* strain B but maintains 16S rRNA sequence identity closer to *C. aggregatum* strain (Fig. 2, Table 3). The phylogenomic analysis shown in Fig. 3 indicates that this strain is the earliest diverging member of the *C. aggregatum* and *C. thermophilum* strains. Collectively, all data suggest strain MS40/45 potentially to be an ecotype or subspecies of *C. aggregatum*.

## Conclusion

We propose that the nine characterized isolates of the genus *Chloracidobacterium* spp. define a new order, *Chloracidobacteriales*, and family, *Chloracidobacteriaceae*, in the class *Blastocatellia* of the phylum *Acidobacteriota*. As previously suggested [41], these nine characterized strains can be assigned to three species: ‘*Ca. C. thermophilum*’, ‘*Ca. C. aggregatum*’ and *C. validum*. *C. validum* strain BV2-C^T^ is further proposed as the new type species and strain of the genus *Chloracidobacterium* due to the circumstance that the current type species *C. thermophilum* is only effectively and not validly published [32]. *Chloracidobacterium* is proposed as the type genus to designate the family *Chloracidobacteriaceae* and the order *Chloracidobacteriales*.

## Description of *Chloracidobacterium* gen. nov.

Chlor.a.ci.do.bac.te’ri.um. Gr. masc. adj. *chlôros*, greenish-yellow, pale green; N.L. neut. n. *Acidobacterium*, a bacterial genus name; N.L. neut. n. *Chloracidobacterium*, a green *Acidobacterium*.

The description is based on that of ‘*Chloracidobacterium*’ Tank and Bryant 2015 [32]. Microaerophilic, oligotrophic, moderately thermophilic, anoxygenic, chlorophotoheterotrophic eubacterium. Cells stained Gram-negative were non-motile and rods that divide by binary fission. Contain chlorosomes with BChl *c* as light-harvesting organelles combined with a BChl *a*-binding Fenna–Matthews–Olson (FMO) protein complex for light energy transfer to homodimeric type-1 reaction centres.

BChl *a* is found in the reaction centres as BChl *a*_P_, Chl *a*_PD_ and Zn-BChl *a*_P_′ as minor Chls [49]. Echinenone, canthaxanthin and *γ*-carotene are the predominant carotenoids. Cells are unable to reduce sulphate and cannot synthesize branched-chain amino acids but can degrade all three. MK-8(H_2_) and MK-8(H_4_) are their major and minor menaquinones, with iso-C_15:0_, C_16:0_, iso-C_16:0_, C_18:0_ and iso-diabolic acid as primary fatty acids. All genus members have DGTA, lyso-DGTA, PE and PME as their predominant polar lipids. Minor amounts of phosphatidyl-*N*,*N*-dimethylethanolamine were also detected, while PG was only present in a few members. The genome comprises two circular chromosomes, and essential genes are found on both. The type species is *C. validum*.

## Description of *Chloracidobacterium validum* sp. nov.

va’li.dum. L. neut. adj. *validum*, valid, worthy, referring to *Chloracidobacterium validum* as the first validly published species name of the genus *Chloracidobacterium*.

Colonies on solid medium are greenish/brownish and lenticular in shape. Cells in liquid medium predominantly occur as greenish-brown aggregates. Optimal irradiance is ~20–50 µmol photons m^−2^ s^−1^ from a tungsten bulb. The DNA G+C content is 59.5 mol% (by sequence). Growth temperature range is 35–50 °C (T_opt_=45 °C) and the pH range is 5.5–9.5 (pH_opt_=7.2) under micro-oxic conditions. Cells cannot reduce sulphate and require supplementation of organic carbon sources. A mix of the common 20 proteinogenic amino acids can serve as carbon, nitrogen and sulphur sources. Growth is possible with all combinations of amino acids or peptone as long as l-leucine, l-isoleucine, l-valine, l-lysine and a reduced sulphur source (e.g. thioglycolate, methionine or cysteine) are provided. Vitamins (i.e. either vitamin B_12_ or a mixture of other vitamins) are essential for growth.

The type strain BV2-C^T^=DSM 113832^T^=JCM 39534^T^ was isolated from a microbial mat collected in Rupite hot springs, Bulgaria (41° 27′ 28″ N 23° 15′ 43″ E [41]; see Fig. S1 and Strunecký *et al.* [44] for a more detailed description of Rupite hot spring). The DNA G+C content of strain BV2-C^T^ is 59.5 mol%. The 16S rRNA and whole-genome sequences of strain BV2-C^T^ are available in GenBank/EMBL/DDBJ under OR946236, CP072648 and CP072649.

## Description of ‘*Candidatus Chloracidobacterium aggregatum*’

ag.gre.ga’tum. L. neut. part. adj. *aggregatum*, aggregated, clustered, as this species forms cell aggregates when grown in liquid medium.

‘*Ca. C. aggregatum*’ has all general properties of the genus *Chloracidobacterium* (see above). Colonies on solid medium are greenish-brown in colour and lenticular in shape. Cells in liquid medium predominantly occur as greenish-brown aggregates. Temperature range for growth, ~40–60 °C (optimum ~45–50 °C). Optimal irradiance is ~20–50 µmol photons m^−2^ s^−1^ from a tungsten bulb. Class-Ia ribonucleotide reductase genes (*nrd*AB) are present, but Class-II, cobalamin-dependent ribonucleotide reductase genes are absent (except strain MS 40/45).

The proposed nomenclatural type is the genome sequence of strain E, represented by GenBank/EMBL/DDBJ accession numbers CP072644 (Chromosome 1) and CP072645 (Chromosome 2). Strain E was isolated from a microbial mat associated with Mushroom Spring in the Lower Geyser Basin of YNP, WY, USA (NPS Scientific Research and Collecting Permit number #YELL-SCI-0129 [33,41]). Due to the restriction on its availability and the need for a material transfer agreement (MTA) by the U.S. National Park Service, strain E is considered not suitable for valid publication, nor for pro-valid publication (IJSEM Nomenclature Guidelines, https://www.microbiologyresearch.org/content/journal/ijsem?page=about-journal#3). The DNA G+C content is ~62.1 mol%. The 16S rRNA sequence of strain E is available in GenBank/EMBL/DDBJ under KP300943.

## Description of *Chloracidobacteriaceae* fam. nov.

Chlor.a.ci.do.bac.te.ri.a’ce.ae. N.L. neut. n. *Chloracidobacterium*, type genus of the family; - *aceae*, ending to denote family; N.L. fem. pl. n. *Chloracidobacteriaceae*, the family of the genus *Chloracidobacterium*.

The family *Chloracidobacteriaceae* was identified by comparative phylogenetic analysis of 16S rRNA gene sequences and by genomic analyses, which indicated that members of the genus *Chloracidobacterium* form a separate clade (this study; also see [20,32, 34, 41]). Members of this family are chlorophototrophic, microaerophilic, oligotrophic, moderately thermophilic, Gram-stain-negative, non-motile rods that divide by binary fission. Cells employ chlorosomes, the BChl *a*-binding FMO protein for harvesting light energy, and homodimeric type-1 reaction centres to carry out conversion of light energy into chemical energy. Cells synthesize BChl *c*, BChl *a* and Chl *a*; major carotenoids are echinenone and *γ*-carotene, and minor carotenoids include canthaxanthin, deoxyflexixanthin, lycopene and *β*-carotene. Cells are unable to synthesize vitamin B_12_, l-lysine and branched-chain amino acids (l-valine, l-leucine and l-isoleucine) but can degrade branched-chain amino acids. The type genus is *Chloracidobacterium*.

## Description of *Chloracidobacteriales* ord. nov.

Chlor.a.ci.do.bac.te.ri.a’les. N.L. neut. n. *Chloracidobacterium*, type genus of the order; -*ales*, ending to denote an order; N.L. fem. pl. n. *Chloracidobacteriales*, the order of the genus *Chloracidobacterium*.

The order *Chloracidobacteriales* was identified by comparative phylogenetic analysis of 16S rRNA gene sequences, phylogenomic analyses of whole genomic sequences and physiologically distinct traits, i.e. chlorophototrophy, which indicated that the family forms a separate, deeply divergent clade within the class *Blastocatellia* (formerly subdivision 4) in the phylum *Acidobacteriota*. The order *Chloracidobacteriales* is proposed to include a single family, *Chloracidobacteriaceae*, with a single genus, *Chloracidobacterium*, containing three species (‘*Ca. C. thermophilum*’, ‘*Ca. C. aggregatum*’ and *C. validum*). The type genus is *Chloracidobacterium*.

## Emended description of ‘*Candidatus Chloracidobacterium thermophilum*’ Bryant *et al.* 2007

The description is based on that of Bryant *et al.* [28] and Tank and Bryant [32] with the following modifications. Bicarbonate stimulates growth but is not essential. Some strains produce PC. The proposed nomenclatural type is the genome sequence of strain B, represented by GenBank/EMBL/DDBJ accession numbers CP072632 (Chromosome 1) and CP072633 (Chromosome 2). Strain B [32] was isolated from a microbial mat associated with Octopus Spring in the Lower Geyser Basin of YNP, WY, USA (NPS Scientific Research and Collecting Permit number #YELL-SCI-0129 [33, 41]). Due to the restriction on its availability and the need for a material transfer agreement (MTA) by the U.S.National Park Service, strain B is considered not suitable for valid publication, nor for pro-validpublication (IJSEM NomenclatureGuidelines, https://www.microbiologyresearch.org/content/journal/ijsem?page=about-journal# 3). The DNA G+C content is~61.3 mol%.

## Strain availability

*C. validum* strain BV2-C^T^ has been deposited in the open culture collections German Collection for Microorganisms and Cell Cultures and the Japan Collection of Microorganisms (JCM) as DSM 113832 and JCM 39534, respectively. *C. thermophilum* strain B is available from JCM (JCM 30199) as well as the American Type Culture Collection (ATCC), as ATCC BAA-2647. For JCM 30199, an MTA with YNP must be obtained prior to strain acquisition; in the case of ATCC BAA-2647, a ‘Material Transfer Agreement Addendum for National Park Service Products’ is necessary. *C. thermophilum* strain D and *C. aggregatum* strains E, N, S, A, 2 and MS40/45 are available from Dr Vera Thiel upon request under a research MTA from YNP Service, USA.

## Supplementary material

10.1099/ijsem.0.007003 Uncited Fig. S1.

## Abbreviations

AAI: average amino acid identity
ANI: average nucleotide identity
ATCC: American Type Culture Collection
BChl: bacteriochlorophyll
Chl: chlorophyll
CTM: *Chloracidobacterium thermophilum* midnight
dDDH: digital DNA–DNA hybridization
DGTA: diacylglyceryl-hydroxymethyl-*N*,*N*,*N*-trimethyl-beta-alanine
DPG: diphosphatidylglycerol
FMO: Fenna–Matthews–Olson
ICNP: International Code of Nomenclature of Prokaryotes
JCM: Japan Collection of Microorganisms
MAGs: metagenome assembled genomes
MTA: material transfer agreement
NCBI: National Center for Biotechnology Information
NPS: National Park Service
PC: phosphatidylcholine
PE: phosphatidylethanolamine
PG: phosphatidylglycerol
PI: phosphatidylinositol
PME: phosphatidyl-*N*-monomethylethanolamine
TEM: transmission electron microscopy
unid. GPL: unidentified plycophospholipid
YNP: Yellowstone National Park
