# Supplementary material for: Chloracidobacterium validum sp. nov., a thermophilic chlorophotoheterotrophic bacterium of the phylum Acidobacteriota from an alkaline hot spring microbial mat, represents Chloracidobacterium gen. nov., Chloracidobacteriaceae fam. nov. and Chloracidobacteriales ord. nov
Source: Int J Syst Evol Microbiol. 2026 Jan 19;76(1):007003. doi: 10.1099/ijsem.0.007003 (PMC12816986; doi:10.1099/ijsem.0.007003)
Supplement: Uncited Fig. S1. [file ijsem-76-07003-s001.pdf]

Supplementary Material: Text 1, Tables S1-S3 and Figures S1-S6

Chloracidobacterium validum, sp. nov., a thermophilic chlorophotoheterotrophic bacterium of the phylum *Acidobacteriota* from an alkaline hot spring microbial mat, represents *Chloracidobacterium* gen. nov., *Chloracidobacteriaceae* fam. nov. and *Chloracidobacteriales* ord. nov.

Mohit Kumar Saini[1,2,*], Steven B. Kuzyk[3], Cristian Villena-Alemany[2], Sarah Kirstein[3], Jacqueline Wolf[3], Meina Neumann-Schaal[3], Shin Haruta[1], Satoshi Hanada[1], Michal Koblížek[2], Vera Thiel[1,3,4,*], Marcus Tank[1,3,4,*], and Donald A. Bryant[4, 5,†]

[1]Department of Biological Sciences, Tokyo Metropolitan University, 1-1 Minami-Osawa, Hachioji, Tokyo 192-0397, Japan

[2]Laboratory of Anoxygenic Phototrophs, Institute of Microbiology CAS, Třeboň 37901, Czechia

[3]Leibniz Institute DSMZ – German Collection of Microorganisms and Cell Cultures, GmbH Inhoffenstraße 7B 38124 Braunschweig, Germany

[4]Department of Biochemistry and Molecular Biology, The Pennsylvania State University, PA 16802, USA

[5]Department of Chemistry and Biochemistry, Montana State University, Bozeman, MT 59717, USA

[†]**Posthumous authorship:** This publication is dedicated to Dr. Donald A. Bryant, who was a natural scientist by heart and who's working efforts during his more than 50 years in photosynthetic research contributed significantly and changed the view to the physiological, genomic, biochemical and molecular biology of chlorophototrophic bacteria.

***Correspondence:** Mohit Kumar Saini, mohitvtm@gmail.com; Vera Thiel, vera.thiel@dsmz.de; Marcus Tank, marcustank@web.de

Running Title: *Chloracidobacterium validum*, sp. nov.

Article type: Research article; Taxonomic description

Category: New species, *Chloracidobacterium validum*

Supplementary Material Text 1: Composition and preparation of *Chloracidobacterium thermophilum* Midnight Medium (CTM Medium; ATCC Medium 2871 and DSMZ medium 1783)

The CTM Medium consists of two parts. Part one is autoclavable and is called "CTM Medium basis". Part two is named "mixed solution" and contains non-autoclavable components. Solid CTM Medium contains 1% (w/v) Bacto®Agar, which has been washed three times and which is added to the CTM Medium basis prior to autoclaving. When cells are grown in liquid, aliquots of Solutions 5 and/or 6 were repetitively added at three- or four-day intervals to enhance growth. For additional details, see [32-34, 41].

Preparation of 1-liter CTM Medium

1. Combine the following components to create CTM Medium basis (autoclavable):

CTM Medium basis components	/ 1 liter
Solution 1	20 ml
Solution 2	3 ml
Solution 3	2 ml
Solution 4	2.5 ml
HEPES buffer	2.4 g

2. Add ddH_2O close to 1 liter
3. Adjust pH to 7.0 with 2 M KOH
4. Add ddH_2O to 1 liter
5. Autoclave medium 40 min at 121 °C
6. Seal medium bottle immediately after autoclaving to avoid oxygenation
7. Let the medium cool down to around 70 °C
8. Combine the following components in 30 ml of ddH_2O and add the filter sterilized mixture to the autoclaved CTM Medium basis:

Filter-sterilized components	
$NaHCO_3$	0.625 g
Na-Thioglycolate	0.125 g
Solution 5	0.5 ml
Solution 6	10 ml
Vitamin solution A	0.25 ml
Vitamin solution B	0.25 ml

9. Keep CTM Medium tightly sealed and between 50-70˚C prior usage and always use freshly prepared medium.

Preparation of stock solutions

All stock solutions are autoclavable and should be autoclaved before long-term storage at 4-8 °C. Exceptions are stated. ddH_2O is used for all solutions if not mentioned otherwise.

Solution 1 (50x stock) components and amounts per liter

a. $MgSO_4 \bullet 7H_2O$: 3.75 g
b. $CaCl_2 \bullet 2H_2O$: 1.80 g
c. Citric acid: 0.30 g
d. Na-EDTA, pH 8, 0.25 M: 0.60 ml
e. Trace metal solution: 50.0 ml

Solution 2 components and amounts per liter
a. K_2HPO_4: 15.3 g

Solution 3 components and amounts per liter
a. Ferric (NH_4) citrate: 12.0 g

Solution 4 components and amounts per liter
a. 2-Oxoglutarate: 14.6 g

Solution 5 components and amounts per 100 ml
a. Bacto™ Peptone: 10.0 g

Solution 6 components and amounts per 400 ml
a. The 20 proteinogenic amino acids: 100 mg of each amino acid

Trace metal solution components and amounts per liter
a. H_3BO_3: 2.86 g
b. $MnCl_2 \bullet 4H_2O$: 1.81 g
c. $ZnSO_4 \bullet 7H_2O$: 0.222 g
d. $Na_2MoO_4 \bullet 2H_2O$: 0.39 g
e. $CuSO_4 \bullet 5H_2O$: 0.079 g
f. $Co\ (NO_3)_2 \bullet 6H_2O$: 0.0494 g

Vitamin solution A (1000x stock) components and amounts per 100 ml. Dissolve the vitamins in 10mM phosphate buffer, pH7.2. Titrate with NaOH until vitamins are dissolved, filter sterilize afterwards, and freeze in appropriate aliquots until usage.
a. Biotin: 10 mg
b. Riboflavin: 10 mg
c. Thiamine HCl: 100 mg
d. Thiamine pyrophosphate: 100 mg
e. L-Ascorbic acid: 100 mg
f. D-Calcium-pantothenate: 100 mg
g. Folic acid: 100 mg
h. Nicotinamide: 100 mg
j. Nicotinic acid: 100 mg
k. 4-Aminobenzoic acid: 100 mg
l. Pyridoxine HCl: 100 mg
m. Lipoic acid: 100 mg
n. Nicotinamide Adenine Dinucleotide (NAD): 100 mg

Vitamin solution B (1000× stock) components and amounts per 100 ml Dissolve the vitamin B_{12} in water. Titrate with HCl until vitamin B_{12} is dissolved, filter sterilize afterwards, and freeze in appropriate aliquots until usage.
a. Cyanocobalamin (B_{12}): 100 mg

Table S1: Fatty acids and quinones of all *Chloracidobacterium* strains, summarized in Table 2. ND; not detected

Compound	Strain A	Strain B	Strain D	Strain E	Strain N	Strain BV2-C^T	Strain 2	Strain MS40/45
iso-$C_{14:0}$	1.2	2.1	1.2	1.5	1.3	0.8	0.7	1.6
$C_{14:0}$	4.5	3.0	2.6	3.1	3.6	3.9	3.2	3.5
iso-$C_{15:0}$	53.1	40.5	51.6	45.4	39.1	48.5	53.2	35.7
anteiso-$C_{15:0}$	0.4	0.2	0.1	0.3	0.3	0.2	0.2	0.2
$C_{15:0}$	1.5	ND	ND	1.0	1.3	1.3	0.7	1.2
iso-$C_{16:0}$	7.7	13.4	9.5	15.8	7.7	5.5	7.8	12.2
$C_{16:0}$	19.8	21.5	20.6	21.1	27.3	23.0	20.4	28.0
iso-$C_{17:0}$	1.4	2.2	2.0	1.2	0.8	1.4	1.7	1.5
anteiso-$C_{17:0}$	1.1	1.2	ND	1.1	1.1	1.2	0.9	1.1
$C_{17:0}$	ND	ND	ND	ND	0.4	0.7	0.4	0.7
$C_{18:0}$	4.4	10.0	9.6	6.3	13.0	8.7	6.0	9.4
iso-diabolic acid	4.9	6.1	2.8	3.4	4.2	4.7	4.6	5.1
**sum**	100.0	100.0	100.0	100.0	100.0	100.0	100.0	100.0
**Menaquinone (MK)**								
MK-8(H_2)	**98.8**	**100.0**	**99.0**	**99.4**	**98.6**	**98.7**	**98.8**	**98.9**
MK-8(H_4)	**1.2**	**0.0**	**1.0**	**0.6**	**1.4**	**1.3**	**1.2**	**1.1**
**sum**	100	100	100	100	100	100	100	100

Table S2: Polar lipids of all *Chloracidobacterium* strains summarized in Table 2 detected by HPLC-MS analysis. Individual lipid species are described by the total carbon content and saturation degree of their corresponding two fatty acids. **Polar lipid abbreviations:** diacylglyceryl-hydroxymethyl-N,N,N-trimethyl-beta-alanine, DGTA; Phosphatidylethanolamine, PE; Phosphatidyl-N-monomethylethanolamine, PME; Phosphatidyl-N,N-dimethylethanolamine, PDME; Phosphatidylglycerol, PG; unidentified lipid, L.

**Strain**		**A**	**B**	**D**	**E**	**N**	**BV2 -C^T**	**2**	**MS4 0/45**
**DGTA**									
retention time range [min]	21-22								
characteristic fragment [M+H]$^+$ m/z	236.1497								
major species		30:0	30:0	30:0	30:0	30:0	30:0	30:0	30:0
		31:0	31:0	31:0	31:0	31:0	31:0	31:0	31:0
		32:0	32:0	32:0	32:0	32:0	32:0	32:0	32:0
		33:0	33:0	33:0	33:0	33:0	33:0	33:0	33:0
		34:0	34:0	34:0	34:0	34:0	34:0	34:0	34:0
**lyso-DGTA**									
retention time range [min]	28-29.5								
characteristic fragment [M+H]$^+$ m/z	236.1497								
major species		14:0	15:0	16:0	15:0	15:0	16:0	15:0	14:0
		15:0			16:0	16:0			16:0
**PE**									
retention time range [min]	20-21.5								
characteristic neutral loss m/z	141.0191								
major species		29:0	30:0	30:0	30:0	29:0	30:0	29:0	29:0
		30:0	31:0	31:0	31:0			30:0	30:0
					32:0				
**PME**									
retention time range [min]	22-23								
characteristic neutral loss m/z	155.0347								
major species		28:0	29:0	29:0	29:0	28:0	29:0	29:0	29:0
		29:0	30:0	30:0	30:0	30:0		30:0	
		32:0			31:0				
**PDME**									
retention time range [min]	23.5-25								
characteristic neutral loss m/z	168.0426								
major species		31:0	29:0	29:0	30:0	32:0	32:0	32:0	32:0
		32:0	33:0	31:0	32:0				
				32:0					
**PG**									
retention time range [min]	19-20								
characteristic neutral loss m/z	172.0137								
major species		-	-	30:0	-	-	30:0	30:0	-
**L**									
retention time range [min]	21-22								
characteristic neutral loss m/z	162.0528								
major species		30:0	30:0	30:0	30:0	30:0	30:0	30:0	30:0

**Strains**	**A**	**B**	**D**	**E**	**N**	**BV2-C^T**	**2**	**MS40/45**
**Abundant Polar lipids**	**DGTA lyso-DGTA PE, PME**	**DGTA lyso-DGTA PE, PME**	**DGTA lyso-DGTA PE, PME**	**DGTA lyso-DGTA PE, PME**	**DGTA lyso-DGTA PE, PME**	**DGTA lyso-DGTA PE, PME**	**DGTA lyso-DGTA PE, PME**	**DGTA lyso-DGTA PE, PME**
Minor Polar lipids	PDME, L	PDME, L	PDME, PG, L	PDME, L	PDME, L	PDME, PG, L	PDME, PG, L	PDME, L

Table S3: Details of species used to calculate the 16S rRNA phylogenetic tree (Fig. 2), including 16S rRNA gene sequence accession numbers, taxonomic assignments and strain numbers.

16S rRNA Accession No.	Genus, Species and Strain	Taxonomic Group			
		Family	Order	Class	Phylum
KM65878	*Arenimicrobium luteum* Ac_12_G8^T	*Arenimicrobiaceae*	*Blastocatellales*	*Blastocatellia*	*Acidobacteriota*
KP638490	*Brevitalea deliciosa* Ac_16_C4^T	*Arenimicrobiaceae*	*Blastocatellales*	*Blastocatellia*	*Acidobacteriota*
KF840370	*Brevitalea aridisoli* Ac_11_E3^T	*Arenimicrobiaceae*	*Blastocatellales*	*Blastocatellia*	*Acidobacteriota*
KF245634	*Aridibacter famidurans* A22_HD_4H^T	*Blastocatellaceae*	*Blastocatellales*	*Blastocatellia*	*Acidobacteriota*
KF245633	*Aridibacter kavangonensis* Ac_23_E3^T	*Blastocatellaceae*	*Blastocatellales*	*Blastocatellia*	*Acidobacteriota*
KX443571	*Aridibacter nitratireducens* A_24_SHP_-5_238^T	*Blastocatellaceae*	*Blastocatellales*	*Blastocatellia*	*Acidobacteriota*
JQ309130	*Blastocatella fastidiosa* A2-16^T	*Blastocatellaceae*	*Blastocatellales*	*Blastocatellia*	*Acidobacteriota*
KP638491	*Stenotrophobacter namibiensis* Ac_17_F2^T	*Blastocatellaceae*	*Blastocatellales*	*Blastocatellia*	*Acidobacteriota*
KP638489	*Stenotrophobacter roseus* Ac_15_C4^T	*Blastocatellaceae*	*Blastocatellales*	*Blastocatellia*	*Acidobacteriota*
KF840371	*Stenotrophobacter terrae* Ac_28_D10^T	*Blastocatellaceae*	*Blastocatellales*	*Blastocatellia*	*Acidobacteriota*
KP334257	*Tellurimicrobium multivorans* Ac_18_E7^T	*Blastocatellaceae*	*Blastocatellales*	*Blastocatellia*	*Acidobacteriota*
AM749787	*Pyrinomonas methylaliphatogenes* K22^T	*Pyrinomonadaceae*	*Blastocatellales*	*Blastocatellia*	*Acidobacteriota*
OR946236	*Chloracidobacterium validum* BV2-C^T	*Chloracidobacteriaceae*	*Chloracidobacteriales*	*Blastocatellia*	*Acidobacteriota*
KP300945	"*Candidatus* Chloracidobacterium aggregatum" N	*Chloracidobacteriaceae*	*Chloracidobacteriales*	*Blastocatellia*	*Acidobacteriota*
KP300943	"*Candidatus* Chloracidobacterium aggregatum" E	*Chloracidobacteriaceae*	*Chloracidobacteriales*	*Blastocatellia*	*Acidobacteriota*
KP300947	"*Candidatus* Chloracidobacterium aggregatum" 2	*Chloracidobacteriaceae*	*Chloracidobacteriales*	*Blastocatellia*	*Acidobacteriota*
KP300944	"*Candidatus* Chloracidobacterium aggregatum" A	*Chloracidobacteriaceae*	*Chloracidobacteriales*	*Blastocatellia*	*Acidobacteriota*
KP300946	"*Candidatus* Chloracidobacterium aggregatum" S	*Chloracidobacteriaceae*	*Chloracidobacteriales*	*Blastocatellia*	*Acidobacteriota*
CP072646	"*Candidatus* Chloracidobacterium aggregatum" MS40/45	*Chloracidobacteriaceae*	*Chloracidobacteriales*	*Blastocatellia*	*Acidobacteriota*
KP300942	*Chloracidobacterium thermophilum* D	*Chloracidobacteriaceae*	*Chloracidobacteriales*	*Blastocatellia*	*Acidobacteriota*
CP002514	*Chloracidobacterium thermophilum* B	*Chloracidobacteriaceae*	*Chloracidobacteriales*	*Blastocatellia*	*Acidobacteriota*
AM162405	*Bryobacter aggregatus* MPL3^T	*Bryobacteraceae*	*Bryobacterales*	*Terriglobia*	*Acidobacteriota*
KJ461654	*Paludibaculum fermentans* P105^T	*Bryobacteraceae*	*Bryobacterales*	*Terriglobia*	*Acidobacteriota*
CP000473	"*Candidatus* Solibacter usitatus" Ellin6076	*Bryobacteraceae*	*Bryobacterales*	*Terriglobia*	*Acidobacteriota*
FR666706	*Bryocella elongata* SN10^T	*Acidobacteriaceae*	*Terriglobales*	*Terriglobia*	*Acidobacteriota*
KM083126	*Edaphobacter dinghuensis* DHF9^T	*Acidobacteriaceae*	*Terriglobales*	*Terriglobia*	*Acidobacteriota*

DQ528760	*Edaphobacter modestus* Jbg-1^T	*Acidobacteriaceae*	*Terriglobales*	*Terriglobia*	*Acidobacteriota*
AM887758	*Granulicella paludicola* OB1010^T	*Acidobacteriaceae*	*Terriglobales*	*Terriglobia*	*Acidobacteriota*
AM887757	*Granulicella pectinivorans* TPB6011^T	*Acidobacteriaceae*	*Terriglobales*	*Terriglobia*	*Acidobacteriota*
KX306477	*Pseudacidobacterium ailaaui* PMMR2^T	*Acidobacteriaceae*	*Terriglobales*	*Terriglobia*	*Acidobacteriota*
MF150298	*Paracidobacterium acidisoli* 4G-K13^T	*Acidobacteriaceae*	*Terriglobales*	*Terriglobia*	*Acidobacteriota*
HQ995659	*Occallatibacter riparius* 277 ^{T}	*Acidobacteriaceae*	*Terriglobales*	*Terriglobia*	*Acidobacteriota*
HQ995661	*Occallatibacter savannae* A2-1c^T	*Acidobacteriaceae*	*Terriglobales*	*Terriglobia*	*Acidobacteriota*
KM083127	*Silvibacterium dinghuense* DHOF10^T	*Acidobacteriaceae*	*Terriglobales*	*Terriglobia*	*Acidobacteriota*
KP120761	*Silvibacterium bohemicum* S15^T	*Acidobacteriaceae*	*Terriglobales*	*Terriglobia*	*Acidobacteriota*
AM887760	*Telmatobacter bradus* TPB6017^T	*Acidobacteriaceae*	*Terriglobales*	*Terriglobia*	*Acidobacteriota*
KP120762	*Terracidiphilus gabretensis* S55^T	*Acidobacteriaceae*	*Terriglobales*	*Terriglobia*	*Acidobacteriota*
DQ660892	*Terriglobus roseus* KBS 63^T	*Acidobacteriaceae*	*Terriglobales*	*Terriglobia*	*Acidobacteriota*
KP334258	*Terriglobus albidus* Ac_26_B10^T	*Acidobacteriaceae*	*Terriglobales*	*Terriglobia*	*Acidobacteriota*
FR774763	*Acidicapsa borealis* KA1^T	*Acidobacteriaceae*	*Terriglobales*	*Terriglobia*	*Acidobacteriota*
EU780204	*Acidicapsa ligni* WH120^T	*Acidobacteriaceae*	*Terriglobales*	*Terriglobia*	*Acidobacteriota*
AB561884	*Acidipila rosea* AP8^T	*Acidobacteriaceae*	*Terriglobales*	*Terriglobia*	*Acidobacteriota*
MH396772	*Acidisarcina polymorpha* SBC82^T	*Acidobacteriaceae*	*Terriglobales*	*Terriglobia*	*Acidobacteriota*
MT892922	*Alloacidobacterium dinghuense* 4Y35^T	*Acidobacteriaceae*	*Terriglobales*	*Terriglobia*	*Acidobacteriota*
CP001472	*Acidobacterium capsulatum* ATCC 51196^T	*Acidobacteriaceae*	*Terriglobales*	*Terriglobia*	*Acidobacteriota*
CP000360	"*Candidatus* Koribacter versatilis" Ellin345	"*Candidatus* Korobacteraceae"	*Terriglobales*	*Terriglobia*	*Acidobacteriota*
JX420244	*Thermoanaerobaculum aquaticum* MP-01^T	*Thermoanaerobaculaceae*	*Thermoanaerobaculales*	*Thermoanaerobaculia*	*Acidobacteriota*
KT287072	*Luteitalea pratensis* HEG_-6_39^T	*Vicinamibacteraceae*	*Vicinamibacterales*	*Vicinamibacteria*	*Acidobacteriota*
KP761690	*Vicinamibacter silvestris* Ac_5_C6^T	*Vicinamibacteraceae*	*Vicinamibacterales*	*Vicinamibacteria*	*Acidobacteriota*
AB612241	*Thermotomaculum hydrothermale* AC55^T	*Thermotomaculaceae*	*Thermotomaculales*	*Holophagae*	*Acidobacteriota*
U41563	*Geothrix fermentans* H-5^T	*Holophagaceae*	*Holophagales*	*Holophagae*	*Acidobacteriota*
OP023990	*Geothrix fuzhouensis* SG 12^T	*Holophagaceae*	*Holophagales*	*Holophagae*	*Acidobacteriota*
X77215	*Holophaga foetida* TMBS4^T	*Holophagaceae*	*Holophagales*	*Holophagae*	*Acidobacteriota*
LC505070	*Mesoterricola sediminis* W786^T	*Holophagaceae*	*Holophagales*	*Holophagae*	*Acidobacteriota*
OP984401	*Mesoterricola silvestris* W79^T	*Holophagaceae*	*Holophagales*	*Holophagae*	*Acidobacteriota*
MN908335	*Sulfidibacter corallicola* M133^T	*Acanthopleuribacteraceae*	*Acanthopleuribacterales*	*Holophagae*	*Acidobacteriota*
AB303221	*Acanthopleuribacter pedis* FYK2218^T	*Acanthopleuribacteraceae*	*Acanthopleuribacterales*	*Holophagae*	*Acidobacteriota*
AJ231184	*Gimesia maris* DSM 8797^T	*Planctomycetaceae*	*Planctomycetales*	*Planctomycetia*	*Planctomycetota*

AJ231190	*Rubinisphaera brasiliensis* DSM 5305^T	*Planctomycetaceae*	*Planctomycetales*	*Planctomycetia*	*Planctomycetota*

Table S4: Details of species used to calculate the phylogenomic tree (Fig. 3), including whole genome sequence accession numbers, taxonomic assignments and strain numbers.

Genome Accession No.	Genus, Species and Strain	Family	Order	Class	Family
JAGFIQ010000059	*Aridibacter famidurans* isolate MAG.1 k127_1028782	*Blastocatellaceae*	*Blastocatellales*	*Blastocatellia*	*Acidobacteriota*
NZ_CBXV010000001	*Pyrinomonas methylaliphatogenes* K22^T	*Pyrinomonadaceae*	*Blastocatellales*	*Blastocatellia*	*Acidobacteriota*
CP072648-CP072649	*Chloracidobacterium validum* BV2-C^T	*Chloracidobacteriaceae*	*Chloracidobacteriales*	*Blastocatellia*	*Acidobacteriota*
JBDNDF000000000	"*Ca.* Chloracidobacterium" sp. mat red bins.4	*Chloracidobacteriaceae*	*Chloracidobacteriales*	*Blastocatellia*	*Acidobacteriota*
JBDNCT000000000	"*Ca.* Chloracidobacterium" sp. mat yellow bins.13	*Chloracidobacteriaceae*	*Chloracidobacteriales*	*Blastocatellia*	*Acidobacteriota*
JBDNCY000000000	"*Ca.* Chloracidobacterium" sp. mat green bins.5	*Chloracidobacteriaceae*	*Chloracidobacteriales*	*Blastocatellia*	*Acidobacteriota*
CP072642-CP072643	"*Ca.* Chloracidobacterium aggregatum" N	*Chloracidobacteriaceae*	*Chloracidobacteriales*	*Blastocatellia*	*Acidobacteriota*
CP072644-CP072645	"*Ca.* Chloracidobacterium aggregatum" E	*Chloracidobacteriaceae*	*Chloracidobacteriales*	*Blastocatellia*	*Acidobacteriota*
CP072636-CP072637	"*Ca.* Chloracidobacterium aggregatum" 2	*Chloracidobacteriaceae*	*Chloracidobacteriales*	*Blastocatellia*	*Acidobacteriota*
CP072640-CP072641	"*Ca.* Chloracidobacterium aggregatum" A	*Chloracidobacteriaceae*	*Chloracidobacteriales*	*Blastocatellia*	*Acidobacteriota*
CP072638-CP072639	"*Ca.* Chloracidobacterium aggregatum" S	*Chloracidobacteriaceae*	*Chloracidobacteriales*	*Blastocatellia*	*Acidobacteriota*
CP072646-CP072647	"*Ca.* Chloracidobacterium aggregatum" MS40/45	*Chloracidobacteriaceae*	*Chloracidobacteriales*	*Blastocatellia*	*Acidobacteriota*
CP072634-CP072635	*Chloracidobacterium thermophilum* D	*Chloracidobacteriaceae*	*Chloracidobacteriales*	*Blastocatellia*	*Acidobacteriota*
CP072632-CP072633	*Chloracidobacterium thermophilum* B	*Chloracidobacteriaceae*	*Chloracidobacteriales*	*Blastocatellia*	*Acidobacteriota*
NZ_CP063849	*Paludibaculum fermentans* P105^T	*Bryobacteraceae*	*Bryobacterales*	*Terriglobia*	*Acidobacteriota*
NZ_JNIF01000001	*Bryobacter aggregatus* MPL3^T	*Bryobacteraceae*	*Bryobacterales*	*Terriglobia*	*Acidobacteriota*
CP000473	"*Ca.* Solibacter usitatus" Ellin6076	*Bryobacteraceae*	*Bryobacterales*	*Terriglobia*	*Acidobacteriota*
JACDQQ010000001	"*Ca.* Acidiferrum panamense" Pan2503	"*Ca.* Acidiferrum"	"*Ca.* Acidoferrales"	*Terriglobia*	*Acidobacteriota*
NZ_JAGSYI010000010	*Acidicapsa acidisoli* SK-11^T	*Acidobacteriaceae*	*Terriglobales*	*Terriglobia*	*Acidobacteriota*
NZ_JAGSYH010000010	*Acidicapsa dinghuensis* 4GSKX^T	*Acidobacteriaceae*	*Terriglobales*	*Terriglobia*	*Acidobacteriota*
NZ_JAGSYG010000010	*Acidicapsa ligni* WH120^T	*Acidobacteriaceae*	*Terriglobales*	*Terriglobia*	*Acidobacteriota*
NZ_SDMK01000001	*Acidipila dinghuensis* DHOF10^T	*Acidobacteriaceae*	*Terriglobales*	*Terriglobia*	*Acidobacteriota*
NZ_SMGK01000001	*Acidipila rosea* AP8^T	*Acidobacteriaceae*	*Terriglobales*	*Terriglobia*	*Acidobacteriota*
NZ_CP030840	*Acidisarcina polymorpha* SBC82^T	*Acidobacteriaceae*	*Terriglobales*	*Terriglobia*	*Acidobacteriota*
NC_012483	*Acidobacterium capsulatum* 161^T	*Acidobacteriaceae*	*Terriglobales*	*Terriglobia*	*Acidobacteriota*
NZ_CP060394	*Alloacidobacterium dinghuense* 4Y35^T	*Acidobacteriaceae*	*Terriglobales*	*Terriglobia*	*Acidobacteriota*
NZ_FNVA01000014	*Bryocella elongata* SN10^T	*Acidobacteriaceae*	*Terriglobales*	*Terriglobia*	*Acidobacteriota*
RSDW01000001	*Edaphobacter aggregans* EB153^T	*Acidobacteriaceae*	*Terriglobales*	*Terriglobia*	*Acidobacteriota*
NZ_JQKI01000001	*Edaphobacter aggregans* Wbg-1^T	*Acidobacteriaceae*	*Terriglobales*	*Terriglobia*	*Acidobacteriota*
NZ_JAGSYF010000010	*Edaphobacter bradus* 4MSH08^T	*Acidobacteriaceae*	*Terriglobales*	*Terriglobia*	*Acidobacteriota*
NZ_JAGSYJ010000010	*Edaphobacter dinghuensis* DHF9^T	*Acidobacteriaceae*	*Terriglobales*	*Terriglobia*	*Acidobacteriota*

NZ_CP073697	*Edaphobacter flagellatus* HZ411^{T}	*Acidobacteriaceae*	*Terriglobales*	*Terriglobia*	*Acidobacteriota*
NZ_SHKW01000001	*Edaphobacter modestus* Jbg-1^{T}	*Acidobacteriaceae*	*Terriglobales*	*Terriglobia*	*Acidobacteriota*
JAGSYE010000010	*Granulicella aggregans* TPB6028^{T}	*Acidobacteriaceae*	*Terriglobales*	*Terriglobia*	*Acidobacteriota*
NZ_JAGTUT010000001	*Granulicella arctica* MP5ACTX2^{T}	*Acidobacteriaceae*	*Terriglobales*	*Terriglobia*	*Acidobacteriota*
NZ_FOZL01000003	*Granulicella pectinivorans* TPB6011^{T}	*Acidobacteriaceae*	*Terriglobales*	*Terriglobia*	*Acidobacteriota*
FZOU01000024	*Granulicella rosea* T4^{T}	*Acidobacteriaceae*	*Terriglobales*	*Terriglobia*	*Acidobacteriota*
NZ_RDSM01000010	*Granulicella sibirica* AF10^{T}	*Acidobacteriaceae*	*Terriglobales*	*Terriglobia*	*Acidobacteriota*
NC_015064	*Granulicella tundricola* MP5ACTX9^{T}	*Acidobacteriaceae*	*Terriglobales*	*Terriglobia*	*Acidobacteriota*
NZ_CP121196	*Telmatobacter* sp. DSM 110680	*Acidobacteriaceae*	*Terriglobales*	*Terriglobia*	*Acidobacteriota*
NZ_LAIJ01000001	*Terracidiphilus gabretensis* S55^{T}	*Acidobacteriaceae*	*Terriglobales*	*Terriglobia*	*Acidobacteriota*
NZ_JAGTAS010000008	*Terriglobus albidus* Ac_26_B10^{T}	*Acidobacteriaceae*	*Terriglobales*	*Terriglobia*	*Acidobacteriota*
NZ_CP042806	*Terriglobus albidus* ORNL^{T}	*Acidobacteriaceae*	*Terriglobales*	*Terriglobia*	*Acidobacteriota*
NZ_JAGSYB010000001	*Terriglobus aquaticus* 03SUJ4^{T}	*Acidobacteriaceae*	*Terriglobales*	*Terriglobia*	*Acidobacteriota*
NC_018014	*Terriglobus roseus* KBS63^{T}	*Acidobacteriaceae*	*Terriglobales*	*Terriglobia*	*Acidobacteriota*
CP002467	*Terriglobus saanensis* SP1PR4^{T}	*Acidobacteriaceae*	*Terriglobales*	*Terriglobia*	*Acidobacteriota*
NZ_JAGSYA010000001	*Terriglobus tenax* DRP35^{T}	*Acidobacteriaceae*	*Terriglobales*	*Terriglobia*	*Acidobacteriota*
CP093313	*Occallatibacter riparius* 277^{T}	*Acidobacteriaceae*	*Terriglobales*	*Terriglobia*	*Acidobacteriota*
NZ_QVQT02000001	*Paracidobacterium acidisoli* 4G-K13^{T}	*Acidobacteriaceae*	*Terriglobales*	*Terriglobia*	*Acidobacteriota*
NZ_JIAL01000001	*Pseudacidobacterium ailaaui* PMMR2^{T}	*Acidobacteriaceae*	*Terriglobales*	*Terriglobia*	*Acidobacteriota*
NZ_JACHEK010000001	*Silvibacterium bohemicum* S15^{T}	*Acidobacteriaceae*	*Terriglobales*	*Terriglobia*	*Acidobacteriota*
NC_008009	"*Candidatus* Koribacter versatilis" Ellin345	"*Ca.* Korobacteraceae"	*Terriglobales*	*Terriglobia*	*Acidobacteriota*
NZ_CP015136	*Luteitalea pratensis* HEG_-6_39^{T}	*Vicinamibacteraceae*	*Vicinamibacterales*	*Vicinamibacteria*	*Acidobacteriota*
JADKCH010000001	"*Ca.* Geothrix odensensis" OdNE_18-Q3-R46-58_MAXAC.008	*Holophagaceae*	*Holophagales*	*Holophagae*	*Acidobacteriota*
JADKIO010000001	"*Ca.* Geothrix skivensis" Skiv_18-Q3-R9-52_MAXAC.067	*Holophagaceae*	*Holophagales*	*Holophagae*	*Acidobacteriota*
JAKZLE010000001	*Geothrix alkalitolerans* SG263^{T}	*Holophagaceae*	*Holophagales*	*Holophagae*	*Acidobacteriota*
JANHMP010000001	*Geothrix campi* SG10^{T}	*Holophagaceae*	*Holophagales*	*Holophagae*	*Acidobacteriota*
BSDC01000001	*Geothrix edaphica* Red802^{T}	*Holophagaceae*	*Holophagales*	*Holophagae*	*Acidobacteriota*
NZ_KE386810	*Geothrix fermentans* H-5^{T}	*Holophagaceae*	*Holophagales*	*Holophagae*	*Acidobacteriota*
JANHMQ010000001	*Geothrix fuzhouensis* SG12^{T}	*Holophagaceae*	*Holophagales*	*Holophagae*	*Acidobacteriota*
BSDE01000001	*Geothrix limicola* Red804^{T}	*Holophagaceae*	*Holophagales*	*Holophagae*	*Acidobacteriota*
JAKZLD010000001	*Geothrix mesophila* SG198^{T}	*Holophagaceae*	*Holophagales*	*Holophagae*	*Acidobacteriota*
AP027079	*Geothrix oryzae* Red222^{T}	*Holophagaceae*	*Holophagales*	*Holophagae*	*Acidobacteriota*
JAKZLB010000001	*Geothrix oryzisoli* SG189^{T}	*Holophagaceae*	*Holophagales*	*Holophagae*	*Acidobacteriota*
JAKZLC010000001	*Geothrix paludis* SG195^{T}	*Holophagaceae*	*Holophagales*	*Holophagae*	*Acidobacteriota*
BSDD01000001	*Geothrix rubra* Red803^{T}	*Holophagaceae*	*Holophagales*	*Holophagae*	*Acidobacteriota*

Supplementary Material: Text 1, Tables S1-S3 and Figures S1-S6

JAKZLA010000001	*Geothrix terrae* SG184^{T}	*Holophagaceae*	*Holophagales*	*Holophagae*	*Acidobacteriota*
NZ_KI912268	*Holophaga foetida* TMBS4^{T}	*Holophagaceae*	*Holophagales*	*Holophagae*	*Acidobacteriota*
NZ_AP027081	*Mesoterricola sediminis* W786^{T}	*Holophagaceae*	*Holophagales*	*Holophagae*	*Acidobacteriota*
NZ_AP027080	*Mesoterricola silvestris* W79^{T}	*Holophagaceae*	*Holophagales*	*Holophagae*	*Acidobacteriota*
AP017470	*Thermotomaculum hydrothermale* AC55^{T}	*Thermotomaculaceae*	*Thermotomaculales*	*Holophagae*	*Acidobacteriota*
NZ_JAFREP010000011	*Acanthopleuribacter pedis* FYK2218^{T}	*Acanthopleuribacteraceae*	*Acanthopleuribacterales*	*Holophagae*	*Acidobacteriota*
NZ_CP071793	*Sulfidibacter corallicola* M133^{T}	*Acanthopleuribacteraceae*	*Acanthopleuribacterales*	*Holophagae*	*Acidobacteriota*
NZ_JMFG01000001	*Thermoanaerobaculum aquaticum* MP-01^{T}	*Thermoanaerobaculaceae*	*Thermoanaerobaculales*	*Thermoanaerobaculia*	*Acidobacteriota*
JACXWA010000109	"*Ca.* Sulfomarinibacter kjeldsenii" MAG AM3-C	"*Ca.* Sulfomarinibacteraceae"	*Thermoanaerobaculales*	*Thermoanaerobaculia*	*Acidobacteriota*
JACXVY010000254	"*Ca.* Sulfomarinibacter sp." MAG AM1	*"Ca. Sulfomarinibacteraceae"*	*Thermoanaerobaculales*	*Thermoanaerobaculia*	*Acidobacteriota*
JACXVZ010000143	"*Ca.* Sulfomarinibacter sp." MAG AM2	*"Ca. Sulfomarinibacteraceae"*	*Thermoanaerobaculales*	*Thermoanaerobaculia*	*Acidobacteriota*
NZ_CP043931	*Gimesia maris* DSM 8797^{T}	*Planctomycetaceae*	*Planctomycetales*	*Planctomycetia*	*Planctomycetota*
NC_015174	*Rubinisphaera brasiliensis* DSM 5305^{T}	*Planctomycetaceae*	*Planctomycetales*	*Planctomycetia*	*Planctomycetota*

Supplementary Material: Text 1, Tables S1-S3 and Figures S1-S6

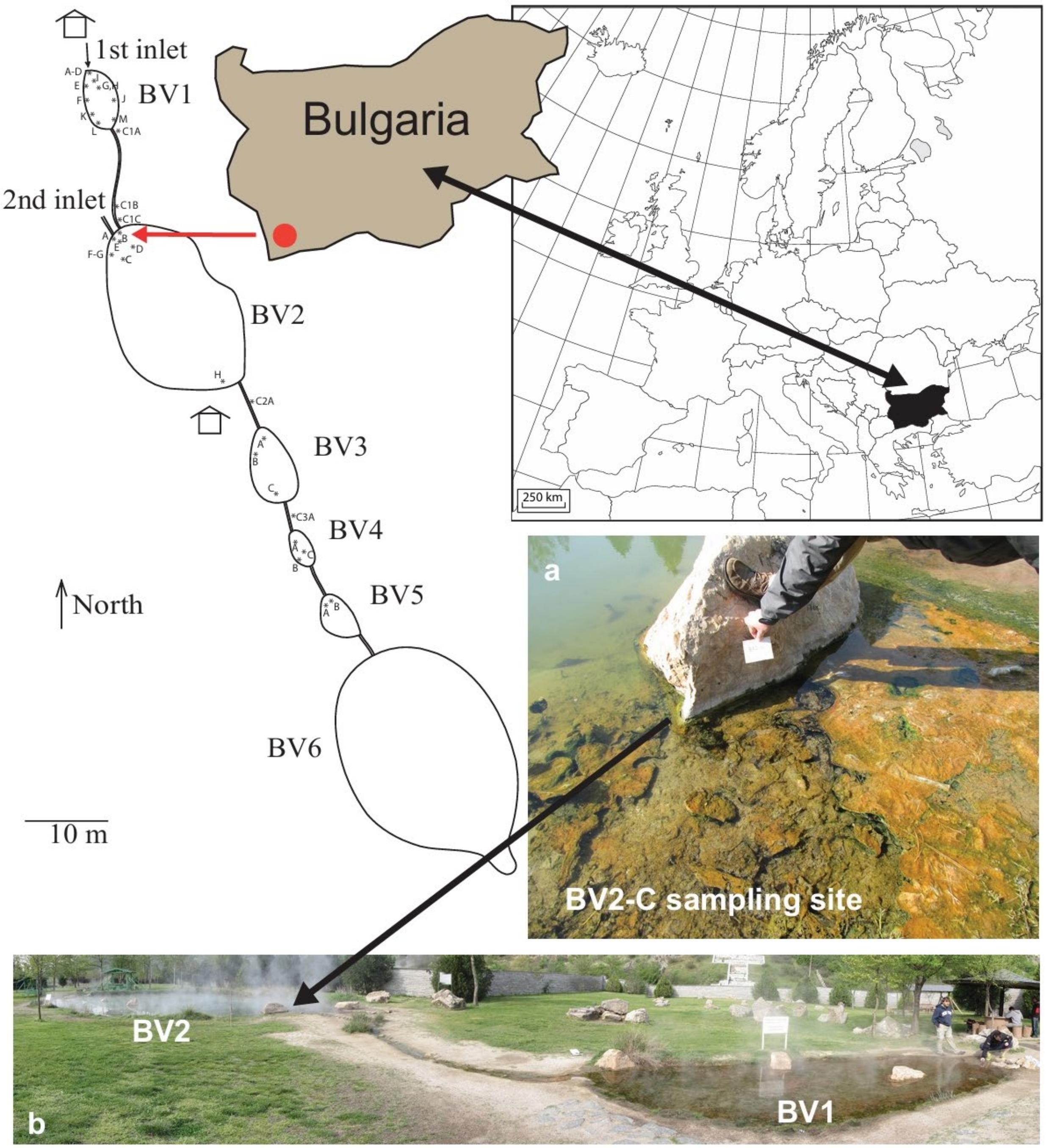

Figure S1. Map and schematic diagram showing the location of Rupite hot spring (red dot; GPS coordinates: 41° 21′ N, 23° 14′ E) in Bulgaria (inset at right showing the location of Bulgaria on the map of Europe). The six pools (BV1 to BV6) of the hot spring cascade from the inlet, where the red arrow indicates an approximate location of sampling site "C" in pool BV2. **(a)** Submerged microbial mats at sampling site BV2-C from which strain BV2-C^T was isolated. **(b)** Pools BV1 and BV2 have a channel connecting them at Rupite hot spring.

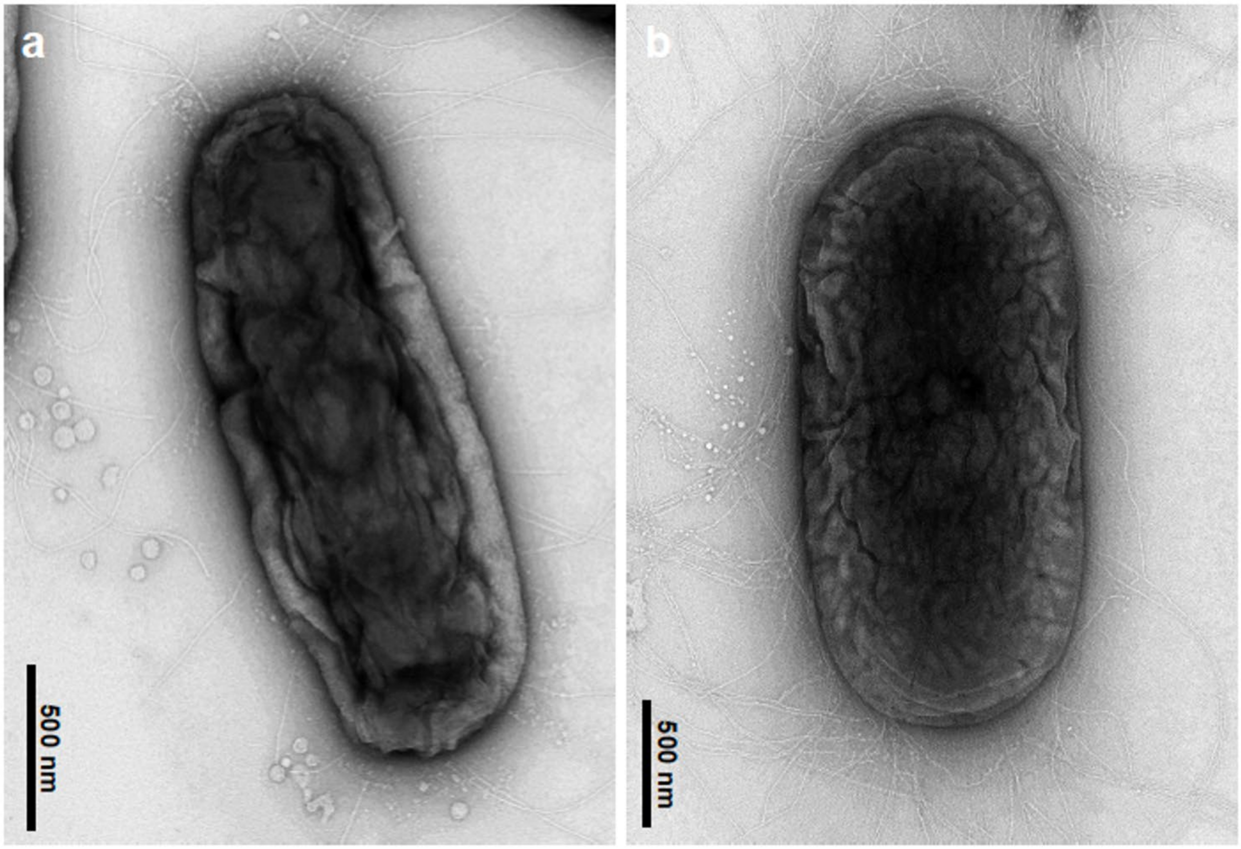

Figure S2. Transmission electron micrographs of (**a**) *C. validum* strain BV2-C^{T} and (**b**) *C. thermophilum* strain B both showing fimbriae/pili. Cells were negatively stained with 1% (w/v) uranyl acetate. Scale bars, 500 nm.

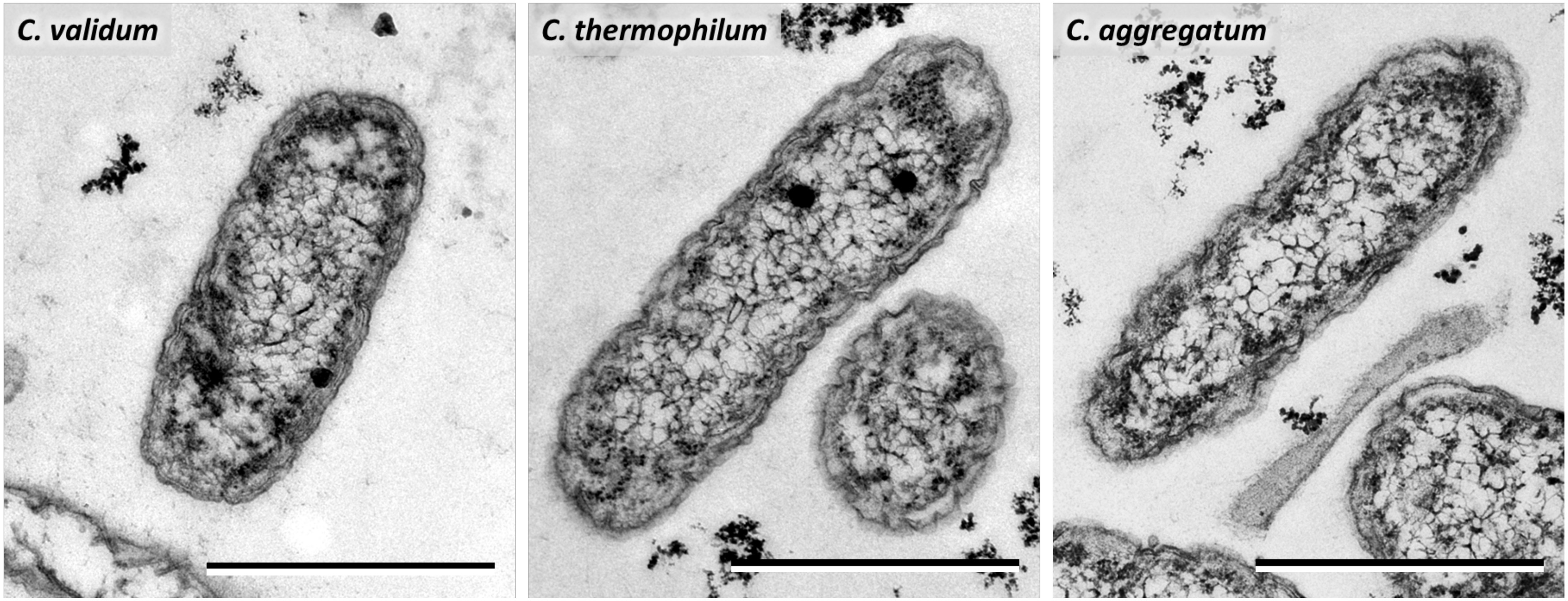

Figure S3. TEM thin sections of *Chloracidobacterium* species. 50 nm thin sections were stained with Lead and Uranylacetate. Gram-negative membranes with peptidoglycan in middle were detected. At the poles of cells of all species small globules are visible. Chlorosomes were not visible at the lateral sides of the cells, as known from TEM images from former experiments of *C. thermophilum* [47]. Scale bar, 1 µm.

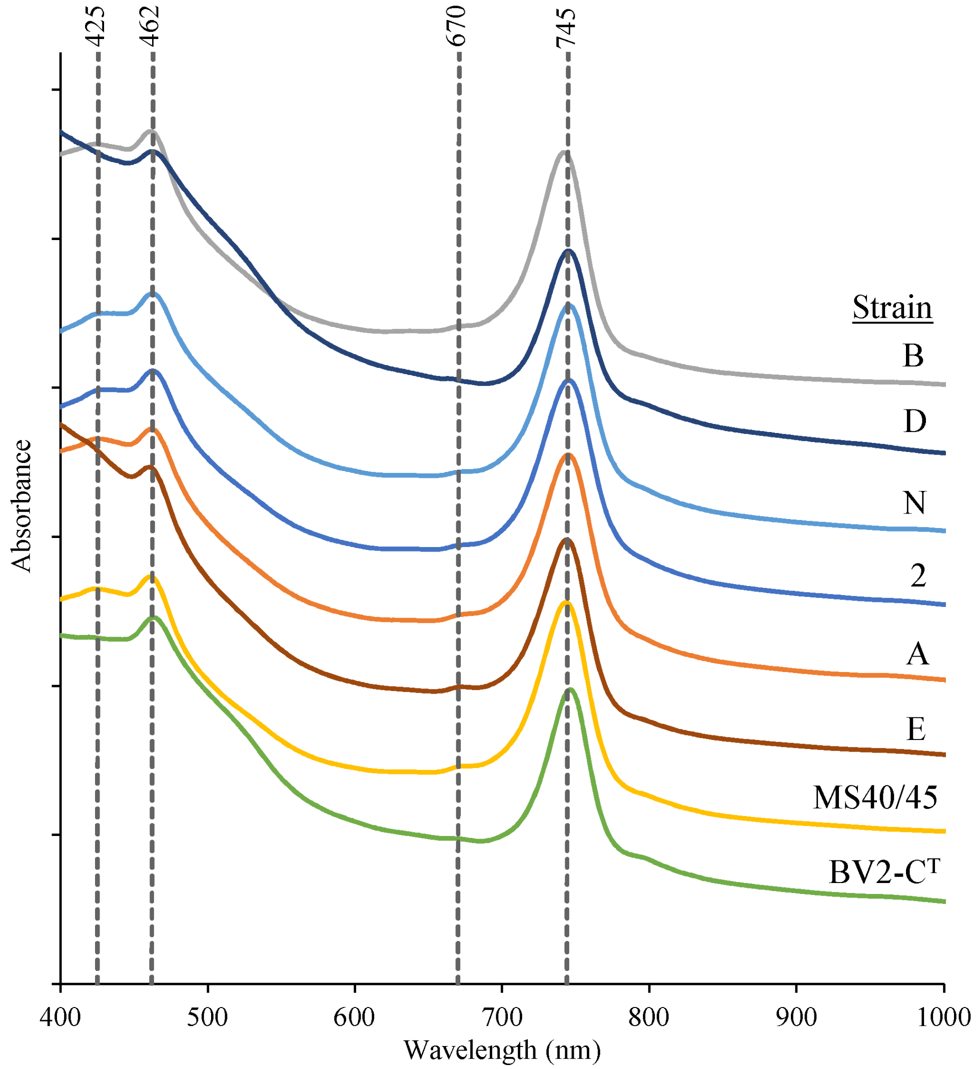

Figure S4. Whole cell absorption spectra of *Chloracidobacterium* strains. Similar maxima shown by vertical dashed lines, suggests all to contain related pigments. Primary reaction center/light harvesting complex maxima for strains B, D, N, 2, A, E, MS40/45 and BV2-C^{T} are 742, 745, 745, 745, 745, 744, 743 and 746 nm, respectively.

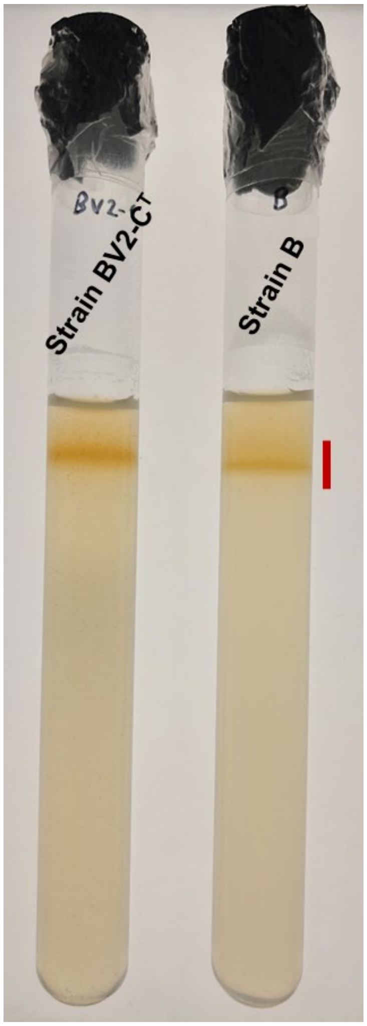

Figure S5. Growth of *C. validum* strain BV2-C^T and *C. thermophilum* strain B in 1% agar deep tubes. Growth in the microoxic zone (2–10 % oxygen) shows the microaerophilic growth of both strains.

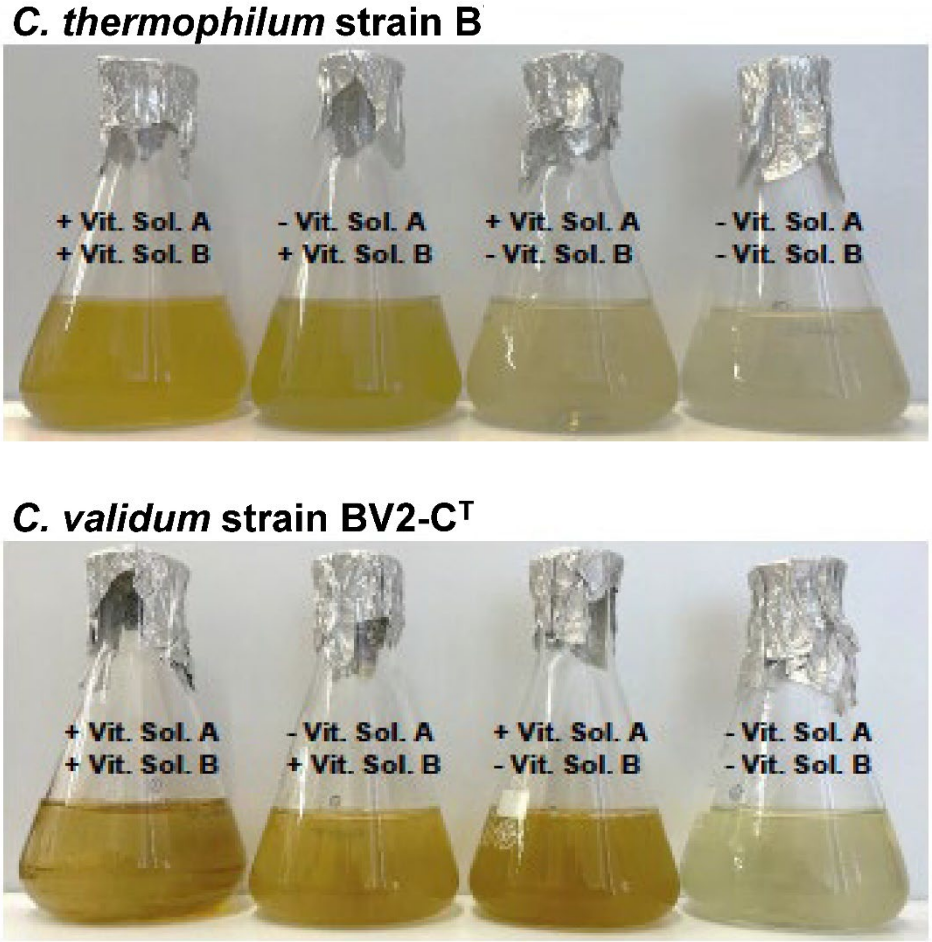

Figure S6. Growth of *C. thermophilum* strain B (upper) and *C. validum* strain BV2-C^T (lower) with different combinations of vitamins as indicated. Vitamin Solution A contained a mixture of 13 vitamins: biotin, riboflavin, thiamine, thiamine pyrophosphate, ascorbic acid, D-calcium-pantothenate, folic acid, nicotinamide, nicotinic acid, 4-amino-benzoic acid, pyridoxine-HCl, lipoic acid, and nicotinamide adenine dinucleotide. Vitamin Solution B contained vitamin B_{12} (cyanocobalamin). The detailed compositions of the vitamin solutions are provided in medium section in the supplemental material text 1. Note that strain BV2-C^T can grow in the absence of vitamin B_{12} but only when vitamin solution A is added, while strain B cannot grow in the absence of vitamin B_{12}.

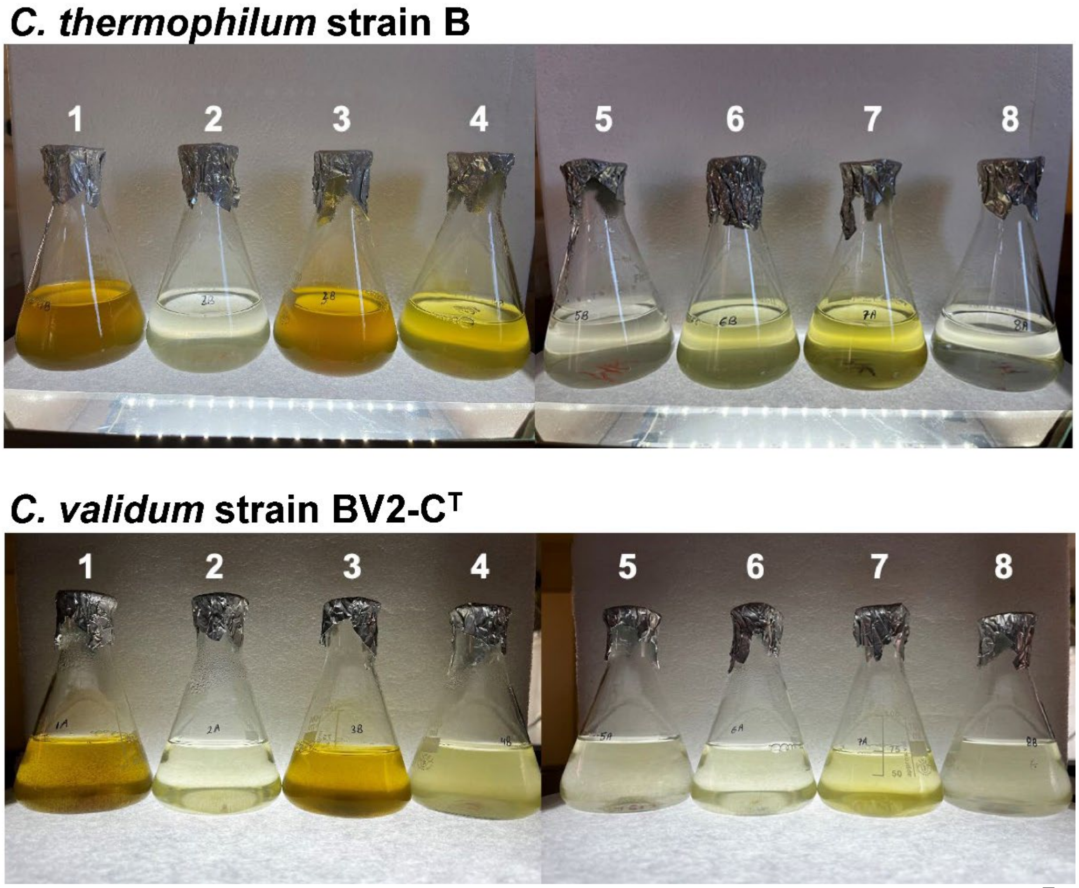

Figure S7. Growth of *C. thermophilum* strain B (upper) and *C. validum* strain BV2-C^T (lower) with different combinations of L-amino acids and/or peptone. Flasks contain: **(1)** 20 amino acids and peptone; **(2)** no amino acids and no peptone; **(3)** 20 amino acids and no peptone; **(4)** only 4 amino acids (L-isoleucine, L-leucine, L-valine and L-lysine) and no peptone; **(5)** only branched-chain amino acids (L-isoleucine, L-leucine and L-valine) and no peptone; **(6)** only L-lysine and no peptone; **(7)** peptone and no amino acids; **(8)** 16 amino acids (except branched chain amino acids (L-isoleucine, L-leucine, and L-valine) and L-lysine) and no peptone. Both strains are obligately dependent on the presence of branched-chain amino acids and L-lysine. Peptone can serve as source of all amino acids, but the growth yield on peptone is lower compared to when individual amino acids were provided.

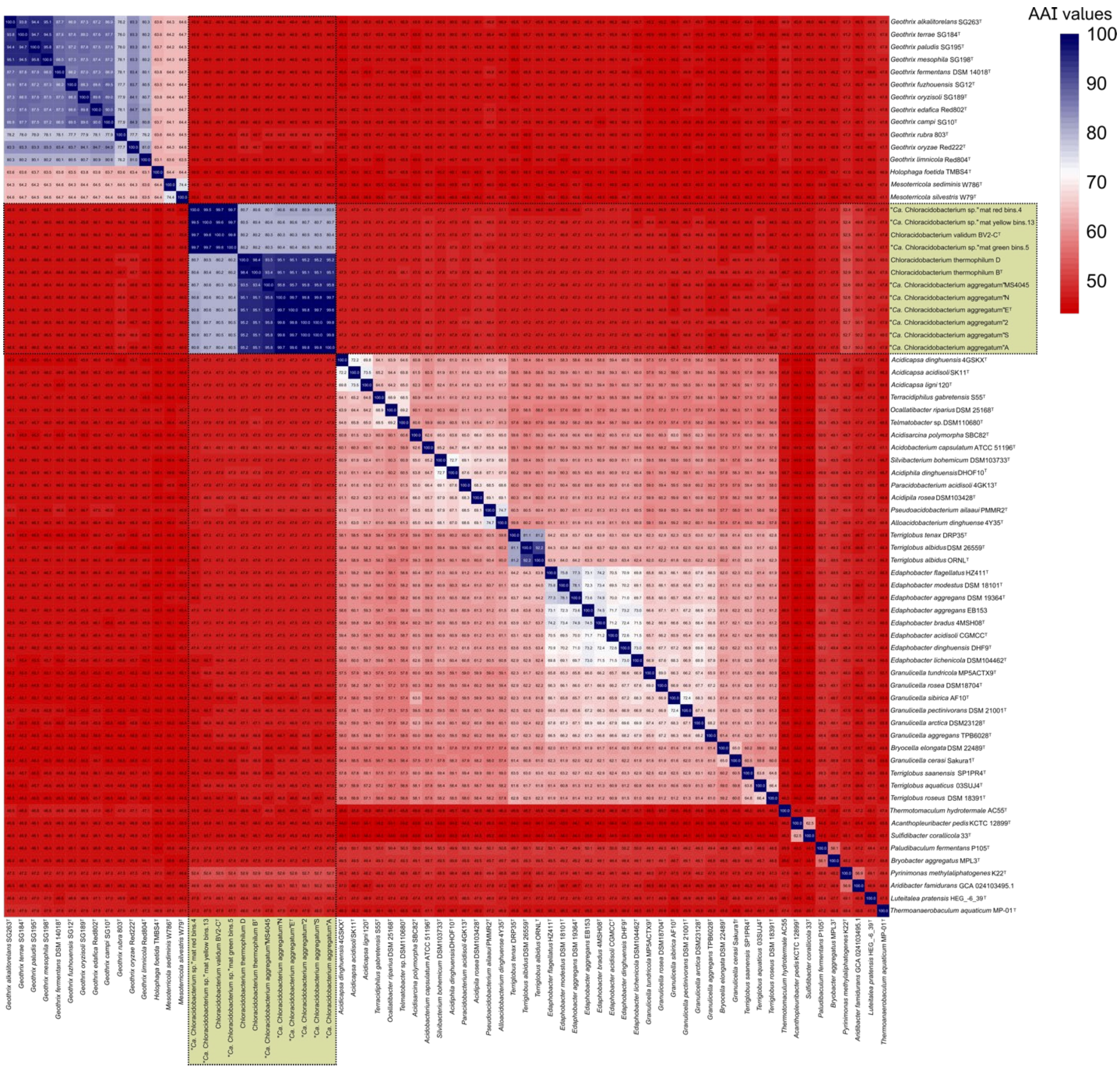

Figure S8. Heat map comparing AAI values for *Chloracidobacterium* spp. strains to other members of the phylum *Acidobacteriota*. AAI values were calculated as described in the main text. All *Chloracidobacterium* spp. strains (names shaded pale green) form a cluster of related strains (upper left quadrant) that is ca. >80 to 99% similar (blue shading surrounded by red) and that is well separated from and only distantly related to all other members of the phylum (red shading).
